# Progress and Perspectives of Molecular Imprinting Methods in the Development of Electrochemical Protein Biosensors

**DOI:** 10.3390/bios16060313

**Published:** 2026-06-01

**Authors:** Suling Yang, Xiaxin Chang, Lin Liu

**Affiliations:** Henan Province of Key Laboratory of New Optoelectronic Functional Materials, College of Chemistry and Chemical Engineering, Anyang Normal University, Anyang 455000, China; yang-sl@aynu.edu.cn (S.Y.); 19837292572@163.com (X.C.)

**Keywords:** electrochemical biosensors, proteins, recognition elements, molecularly imprinting

## Abstract

Protein biomarkers can be used for monitoring the occurrence and development of diseases. Accurate, sensitive, and low-cost methods for protein detection can facilitate therapeutic intervention, improve clinical outcome, and reduce economic pressure for patients. Molecularly imprinted polymers (MIPs) have been considered as a type of biomimetic materials for developing biosensing technologies due to their advantages of high stability, low preparation cost, and good reusability over classical biometric recognition elements such as antibodies and aptamers. Electrochemical biosensors have become the most promising technology in sensing applications in view of their high sensitivity, fast response speed, cost-effectiveness, good stability, and ease of miniaturization. Efforts have been made to develop various electrochemical biosensors for protein detection with MIPs as recognition elements. This article provides an overview of the progress in molecular imprinting methods for the design and application of electrochemical protein biosensors. The strategies for imprinting and removing templates and preparing MIPs-modified sensing electrodes are comprehensively discussed. Finally, the challenges and future perspectives of protein-imprinted electrodes are addressed. This work will contribute to the development of innovative analytical devices based on MIPs for monitoring and managing various diseases by determining protein biomarkers.

## 1. Introduction

Proteins are widely recognized as biomarkers or indicators for monitoring the occurrence and development of various diseases, including infectious diseases, cardiovascular diseases, cancers, and other chronic diseases [1,2]. Accurate, reliable, and sensitive protein-detection technologies are crucial for the early identification of high-risk populations, thereby facilitating timely therapeutic interventions for patients, which is of great significance for improving clinical outcomes and reducing disease burden. Biosensors, which integrate specific biorecognition and signal transduction functions, utilize biometric recognition elements to achieve highly selective quantitative or semi-quantitative analysis of target analytes [3,4,5]. Among various types of biosensors, electrochemical biosensors have become promising technologies in sensing fields owing to their high sensitivity, fast response speed, cost-effectiveness, good stability, small sample size, and ease of miniaturization [6,7]. Such techniques have been commonly applied for determining different biomarkers including proteins, nucleic acids, lipids, etc. However, their practical clinical application still faces many challenges. For example, electrochemical immunosensors based on the antigen-antibody interactions show extremely high selectivity and sensitivity for the assays of human fluids, but their manufacturing process is complex and costly, and environmental factors such as temperature and humidity affect the detection performance [8]. Therefore, developing advanced electrochemical biosensors with integrated merits of reusability, low cost, facile fabrication, high stability, and superior selectivity for the sensitive and specific detection of biomarkers in complex clinical samples is still required.

Molecularly imprinted polymers (MIPs) as a type of biomimetic materials were first proposed by Andersson et al. in the 1980s. Due to their advantages over traditional detection components, such as high stability, low preparation cost, and good reusability, MIPs have received great attention from both the scientific and industrial communities, and are considered ideal materials for developing the next-generation of separation and biosensing technologies [9,10,11,12,13]. MIP-based biosensors have a format similar to the interactions between antibodies and antigens, with specific target recognition abilities. The preparation processes of MIPs typically include three steps. First, monomer–template complexes are formed between the template molecules and the functional monomers through covalent, non-covalent, or ligand exchange reactions. Second, the resulting complexes are polymerized and crosslinked to form three-dimensional polymer matrixes. Third, template molecules are removed from the polymer matrix, leaving vacancies available for target binding. Generally speaking, covalent imprinting and non-covalent imprinting are the two main methods for the preparation of MIPs. Covalent binding is more stable than non-covalent interactions and possesses a more precise stoichiometric relationship, thus enabling higher selectivity and fewer non-specific binding sites. Nevertheless, the imprinting method of covalent binding has the problem of slow template binding and release speed, as this process involves the formation and breaking of covalent bonds. Therefore, in the early stages, non-covalent imprinting is still the most commonly used and flexible method for the design of MIP-based biosensors, as it is easy to operate and has fast template binding and removal speed. However, non-covalent imprinting methods inherently suffer from notable limitations, specifically the inadequate stability of monomer–template complexes and the lack of sufficient precision in their stoichiometric ratios. Under optimal production parameters, the imprinted cavities will effectively preserve the size, shape, function, and various physicochemical properties for template molecules. In this way, the cavities can serve as recognition components for selectively binding targets with the structural similarity to template molecules.

The traditional imprinting methods developed for low molecular weight compounds often fail to address the special challenges posed by large biomolecules such as DNA and proteins [14,15,16,17]. These difficulties mainly stem from the characteristics of biomacromolecules and the following specific reasons. Due to the brittleness of polymers, irreversible structural changes may occur during the polymerization process, making it difficult for MIPs to restore their original structural states. The large volume of biomacromolecules makes it difficult to remove or rebound from MIPs into the polymer networks. Many potential interaction sites on the biomolecule surface and certain characteristics of biomacromolecules may lead to cross reactions caused by non-specific adsorption. All these difficulties have limited the development of protein imprinting technology [18,19]. Although several excellent reviews have discussed the advancement of MIP-based chemosensors and biosensors [20,21,22,23], only limited chapters in them involved electrochemical techniques and protein biomarkers. This work aims to systematically explore different molecular imprinting methods for the design and applicability of electrochemical protein biosensors. The topics of this review mainly focus on the methods for imprinting and removing protein templates and MIP-based electrochemical biosensors for protein detection. Specifically, MIP-based sensing electrodes could be prepared by coating or conjugating pre-synthesized MIP films, nanomaterials, gels, or ionic liquids on the electrode surfaces and by in situ producing imprinting polymers through top-down and bottom-up approaches on the electrode interfaces. Receptor-assisted and epitope-based imprinting methods as well as imprinted self-assembled monolayers (SAMs) for the recognition and capture of proteins are also discussed.

## 2. Methods for Imprinting and Removing Templates

### 2.1. Imprinting Methods

Initially, protein imprinted polymers were prepared by the formation of hydrogels with medium cross-linking ability and large pore size [24]. The obtained hydrogels are then coated on the carrier surface to selectively adsorb proteins. However, proteins are structurally complex and functionally diverse biomolecules, and their numerous functional groups may lead to extensive interactions with various functional monomers. Therefore, proteins are more likely to undergo non-specific binding or cross-reactivity with imprinting polymers. This limits the types of functional monomers and crosslinking reagents that can be used for protein imprinting. Another limiting factor is the difference in physical and chemical properties of different parts of proteins, such as charge and hydrophobicity. Although charged monomers or nanoparticles can serve as an alternative solution to enhance the binding force between proteins and monomers through electrostatic interactions, they may also lead to non-specific binding events. In addition, the charges carried by MIPs may undergo non-specific interactions with the substances that show opposite charges and similar structural domains. The structure flexibility of protein is an additional limiting factor that can be easily adjusted through the changes in chemical composition or temperature. The structural changes of proteins caused by chemical interactions may lead to changes in their basic structure, resulting in the formation of different binding sites and slowing down the remove and rebound process of targets. Considering these factors, efforts have been made to develop new imprinting methods for protein recognition [25].

As an advanced imprinting technique for biomacromolecules, surface imprinting has received widespread attention, especially in the fields of separation and sensing applications [26]. It can effectively reduce the resistance during substance transport and solve the spatial limitations associated with imprinting processes. In this method, the problem of film thickness can be solved through various deposition methods, such as controlled/active radical polymerization, electrochemical deposition of non-conductive polymers, or deposition of SAMs. Monomers can be deposited on conductive substrates to form conductive or non-conductive polymer films through electrochemical polymerization. Insulating polymers exhibit remarkable advantages in the preparation of nanoscale ultra-thin films. When the film thickness exceeds the range of electron transport, usually at the nanometer scale, the insulating layer can prevent the electron transfer between the electrode and the monomer. Meanwhile, a sufficiently dense film will prevent monomers from penetrating into the electrode. This type of biosensor relies on the binding of proteins to the film surface, regulating the permeability of the membrane to redox markers. Signal changes are usually monitored by impedance or voltammetry techniques. Surface imprinting technology for the preparation of MIP-based sensing electrodes can be divided into two approaches: bottom-up and top-down. In the top-down approach, the template is first combined with a certain carrier. After the polymer is formed, the carrier is removed, and the interaction area on the polymer surface will change accordingly. In the bottom-up approach, the template is first fixed onto the substrate, and then the polymer is formed around the template on the substrate. This technology has been used to prepare large molecularly imprinted particles, magnetic nanoparticles, carbon nanotubes, quantum dots (QDs), and sensing electrodes. Fixing the template on the substrate before the polymerization reaction can increase the number of uniform imprinting structures on the polymer surface, thereby improving the performance of MIPs. This template fixation method can be achieved through physical adsorption or covalent bonding interactions.

Physical adsorption is the simplest method for template immobilization, where proteins are attached onto the substrate surface via hydrogen bonds, electrostatic interactions, hydrophobic effects, and van der Waals forces. Common functional monomers include acrylic acid, acrylamide, and 2-hydroxyethyl methacrylate. This approach is mild and can preserve protein structure, with template elution usually achieved by high-ionic-strength solutions, polar solvents, or pH adjustment. In contrast, covalent immobilization offers stable binding and uniform recognition sites. Major covalent strategies include amide bonding via condensation between the carboxyl and amino groups, Schiff-base formation between aldehyde and amino groups, and boronic acid affinity that can specifically recognize cis-diol structures in glycoproteins [27,28,29]. Typical monomers are 4-aminophenylboronic acid, vinylaniline, and glycidyl methacrylate. Template removal relies on acidic conditions, reducing agents, or competitive elution, which greatly improves the specificity and stability of molecularly imprinted electrodes. Among them, boronic acid affinity has been proven to be an efficient method for preparing MIPs to capture glycoproteins. Usually, boronic acid groups are first fixed on the surface of carriers and then react with the sugar chain residues in glycoproteins. Afterwards, polymers can be formed around the proteins to achieve effective imprinting. In order to solve the non-specific adsorption problem caused by the multiple functionalities of imprinting polymers, additional treatment can be carried out to effectively reduce non-specific adsorption. Through this method, a series of MIP materials with high affinity and excellent selectivity have been prepared and used to recognize glycoproteins [28,29].

Epitopes refers to the active sites of small segments located within protein structures in antigens. They can bind to antigen-binding sites on antibodies or lymphocyte receptors. The epitope imprinting method has significant advantages over traditional surface imprinting and bulk imprinting methods [30,31]. For example, it is more suitable for imprinting small segments of proteins, thus avoiding common complexity, size issues, and conformational instability in the imprinting process of biomacromolecules. The small segments in the protein templates can interact with the binding sites of polymers through strong crosslinking agents. The complexity of peptide template is reduced, and its recognition process is similar to a small molecule, which theoretically can improve the binding affinity, selectivity, and specificity. Differing from proteins, peptides lack secondary and tertiary structures and show low sensitivity to the environment. Thus, there is no need to worry about solubility or conformational changes as peptides can be directly dissolved in non-proton organic solvents. Although it is clearly more cost-effective with short peptides as the templates for protein recognition, the design and synthesis of specific peptides may be challenging. For instance, the issue of diffusion limitation still needs to be addressed during the rebinding process of natural targets since the ultimate goal of epitope imprinting is to enable the recognition of whole proteins but not partial fragments.

### 2.2. Removing Methods

During the preparation process of MIPs, the most critical step is the production of corresponding cavities in the polymer matrixes. After the templates are removed by chemical treatment, specific binding sites complementary to the targets will be retained in the polymer matrix. The removal process must effectively remove templates while maintaining the integrity of imprinting structures in the polymers. Insufficient removal may lead to distortion of analytical signals or reduce the recognition ability and selectivity toward targets. In addition, excessively harsh removal conditions may break the binding sites for targets. Currently, the main techniques for removing protein templates include following methods [32]: (1) using reagents such as sodium dodecyl sulfate (SDS), oxalic acid, urea, and ethanol to disrupt hydrophobic interactions and non-covalent bonds; (2) using salts such as NaCl to alter the structure of proteins; (3) using acidic solutions such as sulfuric acid and hydrochloric acid to cleave peptide bonds in protein chains; (4) using alkaline solutions such as sodium hydroxide to denature and aggregate proteins; (5) using enzymes such as trypsin and proteinase K to specifically cleave peptide bonds.

For the removal of protein template, chaotropic agents (commonly referred to as “breaking agents”) are a class of reagents that can specifically destabilize proteins. They can disrupt the hydrogen-bond networks among water molecules, thereby weakening the stability of proteins. Destructing the hydrogen bonds can reduce the hydrophobic effect of proteins, leading to their conformational changes and facilitating their desorption from the imprinting matrix. A wide variety of chemicals can serve as chaotropic agents, including various solvents, certain salts, surfactants, and organic compounds.

Salt treatment is a less commonly employed method for template removal, mainly due to its non-specific disruption of protein structure and generally lower desorption efficiency compared with chaotropic agents, acid and alkali treatments, etc. Salts such as NaCl can disrupt electrostatic forces and hydrogen bonds that maintain the native structure of proteins. Furthermore, a high concentration of NaCl can compete with water molecules surrounding proteins, causing protein dehydration and further structural instability. This process can induce conformational folding of proteins and exposure of hydrophobic sites, thereby facilitating their desorption from the imprinted cavities of MIP and achieving template removal. In addition, NaCl can interact with proteins, promoting their aggregation and conformational change. However, a high concentration of NaCl may cause partial structural collapse of the imprinting matrix, so the salt concentration and treatment time must be strictly controlled in practical applications.

In the above methods, proteins will maintain their original size and primary structure after removal. Another approach for removing protein templates is to decompose proteins into small peptide segments under acidic conditions. Such reactions are usually performed under strong acidic solutions, such as sulfuric acid and hydrochloric acid. In addition, strong acids can protonate amino acid residues in proteins, thereby disrupting electrostatic interactions and hydrogen bonds that maintain the native conformation of proteins. As a result, proteins lose their original structure and function. In some cases, acidic treatment may not achieve satisfactory template removal. Alternatively, other alternative methods can be explored to avoid acidic media. High alkalinity generally induces structural distortion and subsequent aggregation of proteins, as elevated pH can disrupt multiple stabilizing forces within proteins, including hydrogen bonds, deprotonation of amino acid residues, and cleavage of disulfide bonds. Such disruptions allow proteins to unfold and lose their inherent three-dimensional conformation, while the exposed hydrophobic residues are subjected to aggregation, minimizing contact with the surrounding solvents. Additionally, alkaline environments can facilitate protein denaturation and precipitation, further reinforcing aggregation. Both of these effects can be effectively exploited to accomplish efficient protein template removal.

Although protease-assisted protein template removal is relatively uncommon, it possesses high specificity and causes minimal damage to the polymer structure, endowing it potential value in the preparation of highly selective MIPs. This method is similar in principle to peptide bond hydrolysis under acidic conditions. The key difference is that strong acid hydrolysis can cleave all peptide bonds non-specifically, whereas proteases exhibit high site-specificity. In the previous studies, protease used for template removal of protein templates mainly included trypsin and proteinase K. Trypsin is a serine protease that can specifically cleave peptide bonds at the carboxyl side of lysine and arginine residues. Proteinase K as a serine protease can cleave peptide bonds at the carboxyl groups of aliphatic or aromatic amino acids. Upon treatment with these two enzymes, proteins can be degraded into short peptides or even individual amino acids.

In addition, electrochemical overoxidation can be used to remove template molecules from porous materials. Usually, this method is only applicable to the porous materials supported by conductive polymers, such as polypyrrole, polyaniline, and poly(o-phenylenediamine) (poly-oPD). Applying an electric potential to the porous material can trigger an oxidation reaction on the electrode surface, altering the polymer structure and discharging it. Consequently, template molecules can be removed because overoxidation destroys specific binding sites for template molecules, allowing them to be released from the porous material. Electrochemical overoxidation is commonly used for small-molecule templates but is rarely applied to protein templates. This is mainly because proteins have large molecular weights and strong binding interactions with MIPs. Overoxidation cannot completely disrupt binding sites and may cause the denatured proteins to adsorb onto the surface of MIPs, thereby decreasing the desorption efficiency.

With the increasing complexity of detection, adopting advanced optimization strategies for the preparation of biosensors has become increasingly important. Introducing machine learning and computational simulation to intelligently optimize polymerization conditions, monomer ratios, reaction parameters, and template removal schemes may achieve high-precision prediction and design of imprinting materials. Some studies evaluate the binding efficiency and compatibility between proteins and conductive polymers through computational methods, in order to improve the performance of biosensors [33,34]. By utilizing advanced computational techniques such as molecular dynamics simulation and predictive modeling, researchers can gain a deeper understanding of the interactions between target proteins and MIPs. Using density functional theory and molecular dynamics simulation methods, the interaction between solvent and monomer can be determined through density functional calculations. Some scholars have optimized electroactive monomers through computational analysis to make them more suitable for protein binding [35,36]. There are also several studies comparing various computational methods for the development of MIPs, including molecular dynamics, molecular mechanics, classical statistical mechanics, and Monte Carlo, Hartree-Fock theory, and semi-empirical as well as density functional theory approaches [16,37]. Each intelligent method has its own scope of application, which depends on the trade-off between speed, accuracy, and computational resources.

## 3. MIP-Based Electrochemical Protein Biosensors

### 3.1. Pre-Synthesized MIP Materials as Electrode Modifiers

Imprinted polymers can effectively promote the MIP–protein interactions and facilitate the diffusion of analytes toward the electrode surface. Two main strategies have been proposed to develop MIP-based electrochemical biosensors. One approach involves the use of MIPs in the form of nanoparticles and the other method adopts thin or ultrathin films [38]. The integration of MIPs with biosensors can be achieved through suitable methods, accompanied by diverse detection techniques. Several modes are available for the development of sensing platforms. MIPs can be pre-synthesized and then modified on a sensing electrode prior to further processing. Alternatively, in situ polymerization can be conducted directly on the sensor surface to synthesize and anchor MIPs in one step. In addition, the combination of imprinting technology and nanotechnology has culminated in the fabrication of nanoscale imprinted materials. In recent years, various methods have been explored for preparing MIP nanoparticles, including precipitation polymerization, emulsion polymerization, core-shell grafting, and solid-phase synthesis [39,40]. Compared with bulk materials, MIP nanoparticles possess a higher surface-to-volume ratio and larger active surface area per unit mass. As a result, template molecules can bind to nanoparticles more easily, improving binding efficiency and facilitating template removal, thereby enhancing overall performance. Although these properties enable wide applications of MIP nanoparticles in imaging, spectrometric analysis, and sample pretreatment, their immobilization onto electrode surfaces must be optimized for sensing applications [41]. This can ensure stable and uniform immobilization of nanoparticles on electrodes while preserving their binding sites for target molecules.

**Scheme 1:**
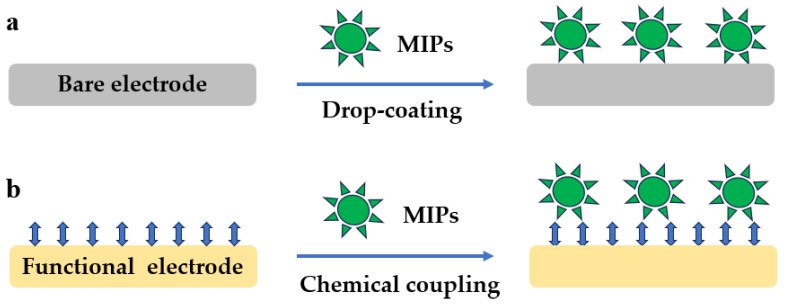
Illustration of drop-coating (**a**) and chemical coupling (**b**) methods for modification of pre-synthesized MIP nanoparticles on the electrode surface.

Two general strategies are commonly adopted during sensor fabrication: MIP nanoparticles are incorporated directly into electrode materials and immobilized on electrode surfaces by using polymer films as supporting scaffolds or grafting suitable linkers such as SAMs onto electrodes (Scheme 1). The first approach can be applied to graphite-based or carbon paste electrodes, in which MIP nanoparticles are simply mixed with other components used for electrode preparation. Although this method facilitates sensor assembly, it cannot guarantee uniform distribution of MIP nanoparticles on the electrode surface. The second method is more flexible and widely applicable, allowing for selection of appropriate immobilization routes based on the properties of MIP nanoparticles to achieve stable attachment. A simple way is to prepare the mixtures of MIP nanoparticles and adhesive materials such as agarose, chitosan, sol-gel materials, or acrylic derivatives (Table 1) [42,43,44,45,46,47,48,49]. These mixtures are then cast onto electrode surfaces via drop-coating or spin-coating. For example, Zhao and co-workers proposed an interesting preparation route using specialized amphiphilic copolymers as the macromonomers with a structure complementary to target proteins including bovine serum albumin (BSA) and ovalbumin (OVA) [43,44]. MIP nanoparticles were obtained by precipitation polymerization of solutions containing the macromonomers and template molecules. The nanoparticle solutions were dropped onto the pretreated gold electrodes and irradiated under UV light for 30 min to achieve UV-cured immobilization. The method showed a linear detection range for BSA from 10^−14^ to 10^−9^ mg/mL.

**Figure 1:**
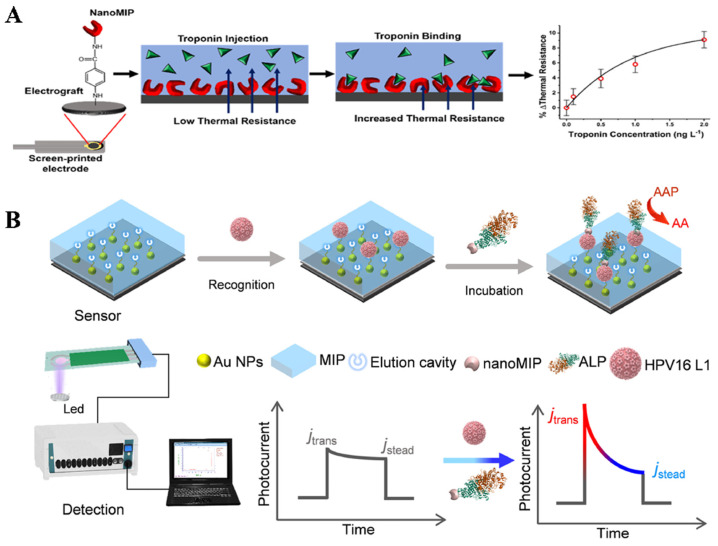
(**A**) Schematic illustration of MIP nanoparticles-modified SPE for cTnI detection and change in thermal resistance with cTnI concentration. Reprinted with permission from ref. [54]. (**B**) Schematic illustration of the HPV16 L1 protein sensing principle adopted by the SDP sensor and change in the photocurrent before and after incubation. Reprinted with permission from ref. [57].

Another method involves the immobilization of pre-synthesized MIP nanoparticles onto electrode surfaces using molecular linkers. Such an approach is generally suitable for gold electrodes, which can be readily labeled with thiol-containing molecules to expose specific functional groups, usually amino or carboxyl groups [50,51,52,53,54,55,56,57]. These groups can react with complementary moieties on MIP nanoparticles, usually through 1-ethyl-3-(3dimethylaminopropyl)-carbodiimide (EDC)/N-hydroxysuccinimides (NHS) coupling chemistry. For example, Garcia-Cruz et al. developed several biosensing platforms to detect diverse targets including small molecules and macromolecular compounds by covalent coupling of MIP nanoparticles on the electrode surface [50]. In their works, MIP nanoparticles were pre-prepared by a solid-phase synthesis method and then covalently attached to screen-printed gold electrodes via sulfur-containing linkers. The electrodes were first treated in cysteamine solution, and MIP nanoparticles were then immobilized on the electrodes through EDC/NHS coupling chemistry. Additionally, Canfarotta et al. reported a similar method for preparing MIP nanoparticles and integrated them into a label-free capacitive biosensor for trypsin detection [53]. To immobilize MIP nanoparticles on gold electrodes, the sensor surface was first subjected to electropolymerization to generate free amino groups. The modified electrodes were then treated with glutaraldehyde solution to crosslink the amino groups on electrodes and MIP nanoparticles. This biosensor achieved a detection limit as low as 10 fM for trypsin. Recently, McClements et al. developed a thermosensitive platform for the detection of cTnI using MIP nanoparticles-functionalized screen-printed graphite electrode (SPE) (Figure 1A) [54]. The nanoparticles were covalently immobilized on the SPE surface through EDC/NHS coupling chemistry and the modified interface was characterized by electrochemical impedance spectroscopy, atomic force microscopy, and scanning electron microscopy. By monitoring the thermal transfer change at the solid–liquid interface, cTnI at the concentration as low as 10 fg/mL has been determined.

In addition, Liu et al. reported a dual-recognition photoelectrochemical biosensor based on a dual-imprinting strategy for the detection of L1 epitope of human papillomavirus type 16 (HPV16) (Figure 1B) [57]. TiO_2_@Au nanostructures were used as the signal amplifiers and MIP nanoparticles labeled with alkaline phosphatase (ALP) served as the secondary identifiers. The ALP-catalyzed hydrolysis of ascorbic acid 2-phosphate into ascorbic acid as an electron donor significantly enhanced the transient photocurrent. Rebinding of target protein limited the generation of steady-state current. The method showed a wide linear range (0.28 pg/mL~28 ng/mL) with a detection limit of 0.1 pg/mL for HPV16 detection.

Soluble nanogels with dimensions comparable to protein clusters have been proven to enable rapid template exchange between polymers and solutions. Interfacial polymerization on supporting particles or electrode surfaces allows the preparation of ultrathin polymer films that only partially cover protein templates. Pre-prepared polymers or solutions containing templates and monomers can be applied onto electrode surfaces via drop coating, spin coating, or dip coating [58,59,60,61,62,63,64,65]. For example, Yang et al. adopted this strategy to fabricate an electrochemical biosensor for BSA detection using MIP nanoparticles pre-prepared by low-temperature polymerization of acrylamide derivatives [63]. A mixture of agarose and MIP nanoparticles was dropped onto glassy carbon electrodes and dried to form a solid film. The sensor exhibited good linear response over a logarithmic concentration range of 10^−16^ to 10^−12^ M. In addition, Wei et al. developed a MIP-based electrochemical biosensor for the detection of BSA in the concentration range from 0.02 to 10 mM with a detection limit of 0.012 mM [65]. The hydrogels were first synthesized under the action of templates through free radical polymerization. The pre-imprinted hydrogels were then drop-cast onto a glassy carbon electrode. The polymerization was allowed to proceed for another 30 min to ensure firm adhesion of the materials onto the electrode surface.

Ionic liquids are a type of molten salts characterized by relatively large ion size and low symmetry. In recent years, ionic liquids have attracted extensive attention in the analysis of biological macromolecules because of their outstanding properties and have become popular subjects in many research fields [66]. Their merits include low volatility with extremely low vapor pressure, wide operating temperature range, high thermal stability, and broad viscosity range. Besides these properties, ionic liquids show wide applicability, good electrolytic conductivity, tunable solubility, non-flammability, and potential reusability. A large variety of ionic liquids can be designed and prepared through combinations of different cations and anions. They can be used as extraction solvents and non-aqueous electrolytes in direct electrochemistry of proteins and serve as the media to effectively improve the efficiency of protein separation. MIPs prepared from vinylimidazolium-based ionic liquid monomers exhibit fast response rates and high selectivity. Other studies have employed ionic liquid monomers to synthesize macroporous polymers in aqueous media with excellent protein adsorption capacity [67,68,69,70,71,72]. For example, Wu et al. reported an electrochemical biosensor for AFP detection using 1-[3-(N-cystamine)propyl]-3-vinylimidazolium tetrafluoroborate ionic liquid [(Cys)VIMBF_4_] to prepare MIP films [72]. Gold nanoparticles were incorporated to further improve the sensing performance of biosensors. The linear detection range was only changed from 0.03 to 5 ng/mL, with a detection limit of 2 pg/mL. Although differential pulse voltammetry was used for quantitative analysis, the performance still could not meet the requirements for clinical application. Such relatively low performance arises from the inherent drawbacks of bulk polymerization, including limited binding capacity and poor selectivity because interactions between the polymer matrix and template molecules are mostly non-specific. In contrast, electropolymerization allows precise control over polymer structure and composition, thereby improving the binding capacity and selectivity of MIPs toward target analytes [67,68,69,70,71].

**Table 1:** Performance of electrochemical biosensors with pre-prepared MIP materials as electrode modifiers for protein detection.

MIP Materials	Elution Reagent	Target	Linear Range	Detection Limit	Ref.
SiO_2_@AgNPs/IDA/EGDMA	methanol/acetic acid (4:1, *v*/*v*)	CEA, CRP	0.1–10^4^ pg/mL	0.046 pg/mL, 0.024 pg/mL	[42]
PAHN copolymer NPs	SDS/acetic acid (10% *w*/*v*:10% *v*/*v*)	BSA, trypsin	0.1–10^10^ fg/mL, 0.1–10^4^ fg/mL	–	[43]
UPDHS	SDS/acetic acid (10% *w*/*v*:10% *v*/*v*)	OVA	10–10^6^ fg/mL	–	[44]
CuFe_2_O_4_	acetonitrile (90%, *v*:*v*)/acetic acid (10%, *v*/*v*)	lysozyme	50–800 ng/mL	1.58 ng/mL	[45]
PDA/GO	1 M HCl/methanol (1:8, *v*/*v*)	BHb	1–10^8^ pg/mL	0.2 pg/mL	[46]
Polyaniline/P(AMPS-co-St)	methanol/acetic acid (19:1, *v*/*v*)	OVA	10–10^6^ fg/mL	1 fg/mL	[47]
Fe_3_O_4_@SiO_2_@DA	SDS/acetic acid	hemoglobin	5–100 μg/mL	1 μg/mL	[48]
Ag-MOF@MC	acetonitrile/acetic acid (1:1 *v*/*v*)	hemoglobin	0.2–10^6^ pM	0.09 pM	[49]
TPIPs/ZnO/Au	SDS/acetic acid	hemoglobin	0.1–10^12^ fg/L	0.031 fg/L	[58]
Alginate gel	100 mg/mL of SDS	CD44	50–10^−7^ fg/mL	14.1 fg/mL	[59]
Alginate gel	10% (*w*/*v*) of SDS	HSA	1–5 × 10^4^ fg/mL	0.03 fg/mL	[60]
CaAlg/CaSiO_3_ hydrogel	Tris-HCl buffer	BSA	30–1.2 × 10^3^ ng/mL	0.23 ng/mL	[61]
Polyacrylamide cryogel	1 M NaCl + 2 g/L SDS	BHb	0.15–10^3^ fM	8.5 aM	[62]
Polyacrylamide cryogel	1 M NaCl + 5 g/L SDS	BSA	0.15–10^3^ fM	7.2 aM	[63]
DTMIPs/Gr-IL	methanol/water (90/10 *v*/*v*)	HSA	0.66–3.6 × 10^3^ ng/mL	15 ng/mL	[67]
Poly(APVIMBF4)/MWCNT	10% (*v*/*v*) acetic acid + 10% (*w*/*w*) SDS	BSA	1.5–1.5 × 10^3^ nM	0.391 nM	[68]
AN/AuNPs/rGO/IL	5% (*v*/*v*) methanol	S protein	0.1–10^3^ ng/mL	38 pg/mL	[69]
Poly(APVIMBr)/MWCNT	0.5 M oxalic acid	myoglobin	0.06–60 μM	9.7 nM	[70]
IL/AuNPs-ZnCdHgSe	NaOH and SDS	HE4	25−4 × 10^3^ pg/mL	15.4 pg/mL	[71]
Poly[(Cys)VIMBF_4_]/AuNPs	10% (*v*/*v*) acetic acid + 10% (*w*/*w*) SDS	AFP	0.03−5 ng/mL	2 pg/mL	[72]

Abbreviation: SiO_2_@AgNPs, silica nanospheres decorated with silver nanoparticles; IDA/EGDMA, iminodiacetic acid/ethylene glycol dimethacrylate polymer; PAHN, electro-polymerizable amphiphilic macro monomer poly(AM-co-HEA-co-NVc); UPDHS, UV-crosslinkable copolymer poly(DMA-co-HEA-co-St); PDA, polydopamine; P(AMPS-co-St), polymer prepared with 2-acrylamido-2-methyl-1-propanesulfonic acid (AMPS) and styrene (St) as monomers; DA, dopamine; Ag-MOF@MC, Ag metal–organic framework mesoporous carbon; TPIPs, thermal responsive protein imprinted polymers; DTMIPs, dual templates molecularly imprinted polymers; Gr-IL, graphene-ionic liquid; APVIMBF4, 3-(3-aminopropyl)-1-vinylimidazolium tetrafluoroborate ionic liquid; MWCNT, multi-walled carbon nanotube; AN, acupuncture needle; AuNPs, gold nanoparticles; rGO, reduced graphene oxide; APVIMBr, 1-{3-[(2aminoethyl)amino]propyl}-3-vinylimidazole bromide ionic liquid; SDS, sodium dodecyl sulfate; CRP, C-reactive protein; BSA, bovine serum albumin; CD44, a transmembrane glycoprotein; HSA, human serum albumin; OVA, ovalbumin; BHb, bovine hemoglobin; HE4, human epididymis protein 4; AFP, alpha-fetoprotein.

### 3.2. In Situ Formation of Imprinting Polymers

**Scheme 2:**
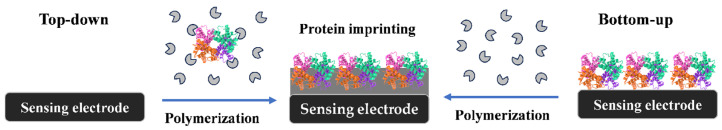
Illustration of top-down and bottom-up approaches for imprinting template proteins.

Free radical polymerization represents a conventional approach for molecular imprinting, in which micron-sized imprinted particles are prepared via procedures such as crushing, grinding, and sieving [73,74,75,76,77]. However, this synthetic method suffers from several limitations for the design of MIP-based electrochemical protein biosensors: uncontrollable particle size of molecularly imprinted materials, irreversible termination reactions, and relatively difficult rebinding between templates and polymers. Surface imprinting is more favorable, allowing protein imprinting on electrode surfaces through photopolymerization, electropolymerization, and other techniques [78,79,80,81,82,83]. Among them, electropolymerization is an effective and straightforward method for fabricating protein-imprinted layers on electrode surfaces. A thin protein-imprinted film can be directly formed by oxidizing electroactive monomers with an oxidizing agent on the electrode interfaces. The advantages of electropolymerization include the precise control of polymer thickness by adjusting the applied potential, which in turn influences charge variations during electrocatalytic processes. The as-prepared protein-imprinted layers exhibit reproducible physical properties such as thickness and porosity, and template removal can be achieved without additional post-treatment of the materials. Generally, electrochemical surface imprinting can be divided into top-down and bottom-up approaches (Scheme 2).

#### 3.2.1. Top-Down Approaches

In the top-down approaches, proteins and functional monomers are mixed in aqueous solvents, followed by electropolymerization. Specifically, protein templates first form complexes with monomers in a non-polar solution, and the complexes are then applied onto the sensor surface via drop-casting. Subsequently, the polymers are crosslinked through electropolymerization, and the protein templates are finally removed by physical or chemical treatment to form the desired cavity structures. Depending on the type of selected monomers and the electropolymerization conditions, either conductive or non-conductive polymer films can be generated. The formation of conductive polymer films is more suitable if the goal is to prepare complex three-dimensional micro- or nanostructured materials, as they favor the fabrication of thicker films. In contrast, the thickness of insulating polymer films is subject to certain limitations. For instance, when the thickness exceeds the range required for electron transport (typically at the nanometer scale), the insulating layer will hinder electron transfer between the electrodes and monomers. Similarly, once the film becomes sufficiently dense, monomers can no longer penetrate toward the electrode surface. At present, cyclic voltammetry is the most commonly used electropolymerization technique for preparing protein-imprinted films. The thickness and compactness of the deposited polymer film can be controlled by adjusting the number of scans and the scan rate. In general, an excessively low scan rate can lead to overly dense films that will prevent effective embedding of template molecules, whereas an excessively high scan rate will result in a loose polymer network, thereby reducing recognition ability. Although potentiostatic deposition cannot precisely control the compactness of the film, it enables accurate control of film thickness by regulating the electric charge consumed during electropolymerization. In addition, the application of pulsed potential can enhance the adhesion between the film and the electrode, facilitating the replenishment of macromolecular templates in the solution layer adjacent to the electrode and thereby promoting better incorporation of protein templates into the growing polymer matrix.

In MIP-based electrochemical biosensors, the electrosynthesis process is influenced by the stacking mode of the monomer layer and the template boundary, which can determine the shape of complementary recognition sites as well as the thickness of polymers. Therefore, the selection of appropriate electroactive functional monomers and the parameters of applied potential range, scan rate, and current density on the electrode surface must all be carefully considered. Conductive polymers are widely used as signal-enhancing materials in electroanalysis, which are usually prepared with phenolic structures of conductive monomers. The polymers can form strongly adherent deposits on the sensor interface to prevent electrode fouling. Phenolic compounds and their derivatives serving as conductive functional monomers mainly include phenol, aminophenol (AMP), dopamine (DA), o-phenylenediamine (oPD), scopoletin, aniline, eriochrome black T, and pyrrole. The monomers can form conductive polymers after electrochemical oxidation by cyclic voltammetry, including polyphenol [84,85,86], polyaminophenol (PolyAMP) [87,88,89], polydopamine (PDA) [90,91,92], poly-oPD [93,94,95,96,97,98,99,100,101], poly(scopoletin) [102,103,104], polyaniline [105,106,107,108], poly(eriochrome black T) [109,110], and polypyrrole [111,112,113,114,115,116,117,118,119,120,121,122,123,124,125,126]. The general potential ranges for the electrochemical oxidation of these monomers with Ag/AgCl as the reference electrode are shown Table 2. The table also exhibits the performance of MIP-based electrochemical biosensors for protein detection. Molecularly imprinted materials based on conductive polymers for protein detection are mainly fabricated by electrochemical oxidation of pyrrole monomers. Prior to the polymerization, the removal of dissolved oxygen from the monomer solution is essential for obtaining stable conductive polymer films, which has been well verified in the preparation of polypyrrole. In addition, the over-oxidation of polypyrrole can be further induced by applying a high potential in buffer solution or using an alkaline solution under aerobic conditions. Aniline and its derivatives, such as AMP and oPD, are also considered ideal candidates for protein imprinting. These compounds possess functional groups capable of participating in various intermolecular interactions including hydrogen bonding and π–π interactions, and the performance depends on the properties of their substituent groups. Apart from favorable biocompatibility, the availability of diverse functional groups and in-depth understanding of the resulting polymers also make DA and acrylamide suitable matrix materials for protein molecular imprinting. The electrochemical transduction mechanisms of these conductive polymers in protein-imprinted polymers have been discussed in the previous reviews [16,25], and the mechanisms of gate-effect and electron transfer phenomenon in both traditional and MIP-based electrochemical biosensors have been thoroughly compared.

**Figure 2:**
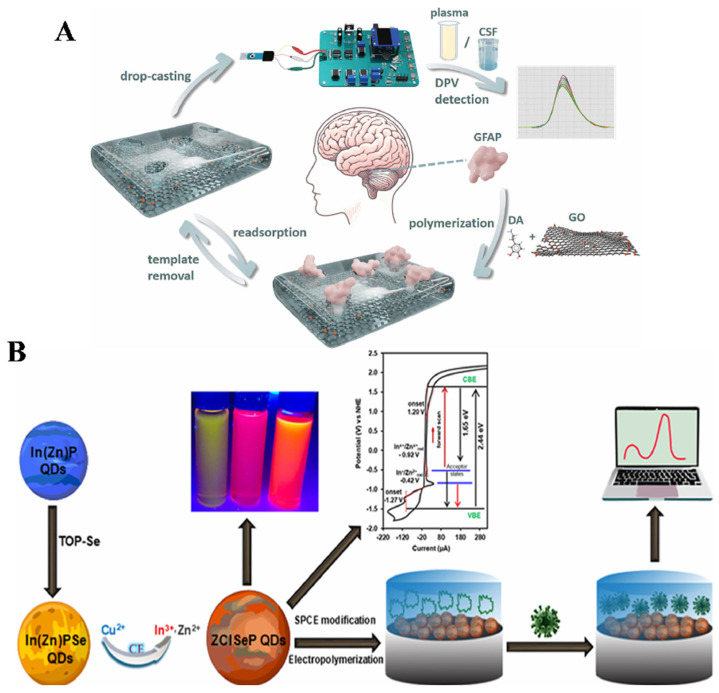
(**A**) Illustration of the synthesis of surface–MIP composite and its application in the detection of GFAP using the home-designed POC readout platform. Reprinted with permission from ref. [92]. (**B**) Schematic illustration of subsequent fabrication of QDs/SPCE by drop casting, polymerization of oPD and nCOV2-S1 protein onto QDs/SPCE, and elution of nCOV2-S1 to form the MIP@QDs/SPCE sensing electrode. Reprinted with permission from ref. [101].

Sensitivity is one of the key parameters for evaluating the practical application value of electrochemical biosensors. In this regard, nanomaterials represent ideal candidates for improving the detection sensitivity due to their unique properties such as large surface area, high electronic conductivity, fast electron transfer rate, and abundant surface-active sites. Many nanomaterials have been proven to be effective transduction mediators in electrochemical biosensors, such as metal nanoparticles, carbon nanomaterials, and QDs. Some of them have been used for the preparation of MIP-based electrochemical protein biosensors, including noble metal nanoparticles (e.g., gold, silver, palladium, platinum), metal oxide nanomaterials (e.g., zinc oxide, magnetite, nickel-cobalt oxide, titanium dioxide), and carbon nanomaterials (e.g., graphene, graphene oxide or GO, reduced graphene oxide or rGO, and carbon nanotubes) [127]. By combining these nanomaterials with MIP technology, the sensitivity and selectivity of electrochemical biosensors for protein detection have been significantly improved (Table 2). More details about the mechanistic roles and properties of nanomaterials used for MIP-based electrochemical biosensors for protein detection can be found in previous professional reviews [38,39,40,41]. The selection of nanomaterials depends on the specific practical application environment and the expected performance of users. For example, carbon nanomaterials as one of the most explored nanomaterials were widely used in molecular imprinting because of their unique properties such as high surface area and good inertness as well as ability to bond with other elements; Li et al. developed an electrochemical platform for the detection of glial fibrillary acidic protein (GFAP) using rGO/PDA molecularly imprinted polymer as the sensing interface (Figure 2A) [92]. The monomer–template complexes were created between DA and GFAP through the formation of hydrogen bonds. The addition of GO and oxidant ammonium persulfate initiated the physical adhesion and polymerization of the complexes to form a PDA–MIP layer. In this method, GO was partially reduced to rGO, and DA was oxidized into quinone followed by the formation of leucodopamine-chrome through intramolecular cyclization. GFAP templates were eluted by 1 mM HCl and 10 mg/mL SDS, producing imprinted cavities with size, shape, surface charge distribution well complementary to the targets. The detection limit of this method is 754.5 ag/mL, which is clinically effective in plasma analysis. The size-dependent tunable electronic and optical characters of QDs make them as idea materials in optoelectronic, electro-optical, photovoltaic, and biomedical applications. MIP nanoparticles can be integrated with QDs and other nanomaterials to enhance the signals, enabling more precise and sensitive detection of targets. Adeniyi et al. achieved the electrochemical detection of SARS-CoV-2-S1 protein in saliva using Zn-Cu-In-Se-P (ZCISeP) QDs-modified electrode (QDs/SPCE) (Figure 2B) [101]. The protein-imprinted polymer film-covered electrode was prepared by surface-imprinting electropolymerization strategy for specific target binding. The rebinding of SARS-CoV-2-S1 to the imprinted cavity modulated the Fe(CN)_6_^3−/4−^ and Cu^1+/2+^ redox processes at the sensor interface and produced a dual on/off proportional current signal. Under optimal conditions, the biosensor exhibited a wide linear detection range (0.001–100 pg/mL) and a low detection limit (0.34 pg/mL).

#### 3.2.2. Bottom-Up Approaches

In the bottom-up approaches, the template is first immobilized on the electrode surface and the carrier is removed after the polymer is formed. This method can increase the number of uniform imprinted structures on the polymer surface and improve the performance of the polymer, and such template immobilization can be achieved through physical adsorption or covalent bonding between the protein and the electrode surface. Physical adsorption is mainly realized through non-covalent forces including electrostatic interaction, hydrophobic interaction, hydrogen bonding, and van der Waals forces, based on the weak interactions between charged groups or hydrophobic regions on the protein surfaces and the electrodes or the modified layers to adsorb and fix the templates (Table 3) [128,129,130,131,132,133,134,135]. This method is mild in operation and can well maintain the native conformation of proteins. The corresponding template removal is mostly carried out via gentle elution using high-ionic-strength salt solutions, polar organic solvents or pH-adjusted buffers, which desorbs the templates by weakening non-covalent interactions without damaging the imprinted cavities.

Nanomaterials are ideal candidates for electrochemical sensor design owing to their large surface area, excellent conductivity, and high catalytic activity. Numerous examples demonstrate that the combination of nanotechnology and imprinting technology can improve the performance of electrochemical biosensors. For this consideration, MIP films have been combined with metallic and carbon-based nanomaterials to take advantage of their superior signal transduction properties. Their multifunctional characteristics including high conductivity, specific surface reactivity, and large surface-to-volume ratio effectively improve detection sensitivity. In addition, for the fabrication of complex 3D microstructures or nanostructured materials, conductive polymer films are more suitable for the preparation of relatively thicker films. At present, voltammetry is the most commonly used polymerization technique for preparing protein-imprinted polymers. The thickness and compactness of the film can be controlled by adjusting the number and rate of potential scanning. Low scanning rates often result in overly dense films, hindering the effective embedding of template molecules. In contrast, excessively high scanning rates will result in loose polymer networks and reduce molecular recognition capabilities. Potentiostatic deposition cannot precisely regulate film compactness, yet it enables accurate control of film thickness by adjusting the charge consumption during the polymerization process. A representative study was reported by Moreira et al., who developed an electrochemical biosensor for troponin T based on nanostructured recognition elements fabricated with the MIP films deposited on multiwalled carbon nanotubes [135]. The nanomaterials were dispersed in a polyvinyl chloride matrix, acting as an adhesive layer covering conductive metal wires named working electrodes. The sensor achieved the detection of troponin T with a detection limit of 160 ng/mL. Rebelo et al. reported a similar study in which MIP films were prepared by copolymerizing multiple monomers in the presence of target molecules immobilized on graphene surfaces [136]. Graphene was oxidized to introduce specific functional groups capable of reacting with activated moieties by EDC/NHS coupling chemistry. The selected charged monomers of positively charged trimethyl-(4-vinylphenyl)ammonium and negatively charged vinyl benzoate could interact with the negatively and positively charged domains of target molecules, respectively. The resulting MIP films exhibited stronger protein recognition ability than that without the use of charged monomers due to enhanced electrostatic interactions with the charged regions of target proteins. The biosensor was fabricated by mixing these imprinted materials with components of solid-state carbon electrodes, enabling protein detection at nanogram per milliliter levels. In addition, Lahcen et al. reported a MIP-based electrochemical biosensor for the detection of human epidermal growth factor receptor 2 (Her-2) protein using laser scribed graphene (LSG) electrodes modified with nanostructured gold to immobilize Her-2 by physical adsorption [128]. After pre-adsorption of the protein template, 3, 4-ethylenedioxythiophene (EDOT) was electropolymerized on the electrode surface by a chronoamperometry technique at 0.85 V for 70 s. The template protein was extracted using ethanol for incubation of 20 min.

Through specially designed interfaces, proteins can be fixed in a defined orientation, maintaining stable conformation and exposing functional regions for efficient recognition. Recently, increasing attention has been paid to oriented or site-specific immobilization of proteins in surface imprinting technology [137]. Covalent bonding allows for the immobilization of proteins by forming stable chemical bonds with high binding strength and can effectively prevent template detachment during polymerization, typically including amide bond, disulfide bond, Schiff-base, and boronic acid affinity (Table 3). Among them, amide bond allows for the immobilization of protein templates through the condensation reactions between amino and carboxyl groups on the proteins and electrode surfaces under the action of activators such as EDC/NHS [136,138,139,140,141,142,143,144]. The templates can be removed by dilute acid or alkali hydrolysis or competitive reagent elution. Tlili et al. reported two surface-imprinted techniques for protein detection through the formation of polydopamine matrix. In the first method, MIPs were prepared by electropolymerization of a mixture of dopamine monomer and IgG template, while in the second method, the protein was pre-immobilized on the mercaptoundecanoic acid (MUDA)-coved electrode surface through EDC/NHS-mediated covalent coupling reaction. The template protein was removed from the vicinity by dipping for 1 h under planar agitation and washing with 0.5 M H_2_SO_4_ and ultrapure water.

Glutaraldehyde-mediated cross-linking can facilitate the immobilization of proteins by forming Schiff base structures between the two aldehyde groups in glutaraldehyde and the amino groups on both the protein and electrode surfaces [145,146,147,148,149,150,151,152]. The templates can be removed by acid–base hydrolysis or competitive elution with amino-containing compounds. For example, Ting et al. achieved the detection of interleukin-6 (IL-6) using the electrode modified with AuNPs and 3-aminopropyltriethoxysilane (APTES) [145]. Glutaraldehyde was used as the linker for protein immobilization and APTES enhanced the peak current through the adsorption with redox probe. The IL-6 template could be removed by NaCl treatment for 120 min. Under optimized conditions, IL-6 has been determined in the concentration range from 2 to 400 pg/mL with a detection limit of 1.74 pg/mL. Disulfide bond formation relies on the reactions between sulfhydryl groups on the protein surface and sulfhydryl or maleimide groups modified on the electrode to form disulfide bonds, which can be broken using reducing agents such as dithiothreitol and β-mercaptoethanol to achieve desorption [153,154,155,156]. Ayankojo et al. reported a MIP-based electrochemical biosensor for the detection of brain-derived neurotrophic factor (BDNF) by functionalization of the thin film metal electrode with 4-aminothiophenol 4-ATP and 3,3-dithiobis(sulfosuccinimidyl propionate) (DTSSP) [153]. DTSSP was used as the linker for the conjugation of BDNF, and meta-phenylenediamine (mPD) was electrodeposited around the template protein to form poly(mPD) film. The removal of protein was carried out by cleaving the disulfide bond of DTSSP with 2-mercaptoethanol and washing the electrode with 10% acetic acid.

**Figure 3:**
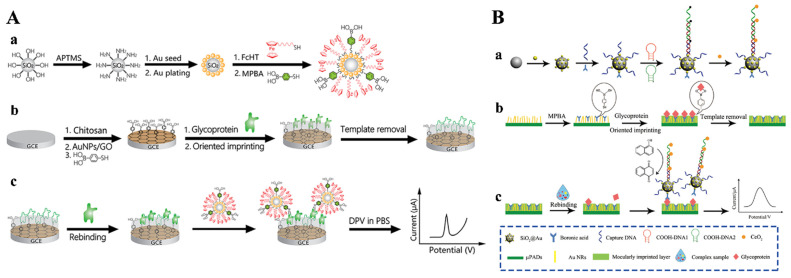
(**A**) Schematic illustration of preparation of SiO_2_@Au/FcHT/MPBA (a), boronate affinity-based glycoprotein-imprinted electrode (b), and sandwich sensing platform for electrochemical detection of glycoproteins (c). Reprinted with permission from ref. [165]. (**B**) Schematic illustration of preparation of the SiO_2_@Au/dsDNA/CeO_2_ signal tag (a), boronate affinity-based glycoprotein imprinted film (b), and sensing platform for electrochemical detection of OVA (c). Reprinted with permission from ref. [167].

In molecularly imprinted polymers, recognition structures matching the shape, size, and functional groups of template molecules can be constructed through various interactions, including covalent bonding, hydrogen bonding, hydrophobic interactions, electrostatic interactions, and van der Waals forces. Since boronic acid can reversibly bind to cis-diol groups of sugar molecules, it serves as an ideal receptor for glycoprotein recognition. This property makes boronic acid serve as a highly valuable ligand in sensing, separation, self-assembly, and other related fields. Several key factors must be considered in glycoprotein imprinting, including the large molecular size and structural complexity of proteins. The imprinting effect is dependent upon the property of imprinting materials, solubility differences between monomers and templates, template purity and availability, as well as the control of imprinted layer thickness [157,158,159,160,161,162,163,164,165,166,167]. You et al. reported a borate affinity-assisted imprinting method for electrochemical detection of glycoproteins (Figure 3A) [165]. The electrode was modified with gold nanoparticles and reduced graphene oxide (AuNPs/GO) to accelerate electron transfer. 4-Vinylphenylboronic acid (VPBA) MIPs were granted on the electrode surface to capture glycoproteins. SiO_2_@Au nanoparticle modified with 6-ferrocenylhexanethiol (FcHT) and 4-mercaptophenylboronic acid (MPBA) (SiO_2_@Au/FcHT/MPBA) was used as the signal label. The sandwich biosensor can detect horseradish peroxidase (HRP) as a model glycoprotein with a linear range of 1 pg/mL ~ 100 ng/mL and a detection limit of 0.57 pg/mL. Sun et al. reported a MPBA/MIP-based microfluidic biosensor for the detection of OVA (Figure 3B) [167]. Gold nanorods were grown on paper and MPBA was assembled on the gold surface to capture OVA. SiO_2_@Au nanocomposite labeled with MPBA and CeO_2_-modified double stranded DNA (SiO_2_@Au/dsDNA/CeO_2_) was used as the signal tag. The nanocomposite can catalyze the oxidation of 1-naphthol to generate an amplified electrochemical signal. The biosensor can determine OVA with a detection limit of 0.87 pg/mL.

**Table 3:** Performances of MIP electrodes prepared with down-top approaches for protein detection.

Immobilization Method	MIP Materials	Elution Reagent	Target	Linear Range	Detection Limit	Ref.
Adsorption	EDOT/LSG-AuNS	ethanol	Her-2	1–200 ng/mL	0.43 ng/mL	[128]
	polyaniline	1 mol/L NaCl	trypsin	0.5–500 ng/mL	5 ng/mL	[129]
	PAP	proteinase K	Myo	0.05–53.3 μg/mL	0.8 μg/mL	[130]
	PAP	oxalic acid	CA 15-3	5–50 U/mL	1.5 U/mL	[131]
	VBTC/CWP	proteinase K	BSA	5–10^5^ μg/mL	0.1 mg/mL	[132]
	APTES-TEOS/PVC	SDS/acetic acid	rhEPO	10–10^3^ ng/mL	6.5 ng/mL	[133]
	PTB	NaOH	PSA	1–60 μg/L	1–60 μg/L	[134]
Amide bond	acrylamide/GO	trypsin	PSA	2–89 ng/mL	2 ng/mL	[136]
	acrylamide	oxalic acid	EGFR, VEGF	0.05–5 × 10^4^ pg/mL, 0.01–7 × 10^3^ pg/mL	0.01 pg/mL, 0.005 pg/mL	[138]
	acrylamide	oxalic acid	PSA, Myo	0.01–100 pg/mL, 1–2 × 10^4^ ng/mL	5.4 pg/mL, 0.83 ng/mL	[139]
	Poly(mPD)	proteinase K	PSA	10–10^8^ pg/mL	–	[140]
	Poly(mPD)	0.5 M H_2_SO_4_	IgG	1–10^13^ fg/L	1 fg/L	[142]
	AMPTMA	proteinase K	CA 15-3	1–10^5^ mU/mL	0.001 U/mL	[143]
	bithiophene	NaOH	HSA	12–300 pM	0.25 pM	[144]
Schiff-base	poly-oPD	NaCl	IL-6	2–400 pg/mL	1.74 pg/mL	[145]
	polyaniline	SDS/acetic acid	BSA	20–2 × 10^5^ pg/mL	2.3 pg/mL	[146]
	polypyrrole/Cu-MOF/chitosan	SDS/acetic acid	IgG	0.01–10 ng/mL	3 pg/mL	[147]
	bithiophene/EG-FET	NaOH	hCG	0.8−50 fM	0.17 fM	[148]
	Scopoletin/Au	SDS/acetic acid	lysozyme	0.15−200 μM	0.9 mg/L	[149]
	poly-oPD/CS/Co-MOF/IL	acetic acid	CEA	0.1−10^4^ pg/mL	0.024 pg/mL	[150]
	AuNPs@rGO	SDS/acetic acid	MAA	0.01−200 ng/mL	5 pg/mL	[151]
	polypyrrole/SiO_2_	HF/oxalic acid	BHb	0.1−10^6^ pg/mL	−	[152]
Disulfide	TFME	MEth + acetic acid	BDNF	10–40 pg/mL	9 pg/mL	[153]
	PAPBA/Au-TFME	DTT + acetic acid	ncovS1	26.7–194 fM	15 fM	[154]
	Poly(mPD)	DTT + acetic acid	HCV E2	0.2–100 pg/mL	0.46 fg/mL	[155]
	Poly(mPD)	DTT/NaCl/DMSO	CDNF	5–50 ng/mL	4.2 ng/mL	[156]
Boronic acid	PAPBA/Cu_7_S_4_-Au	H_2_SO_4_	S protein	5−10^6^ pg/mL	1.76 pg/mL	[157]
	3-TBA/pTH/pABSA	HCl	OVA	1–10^5^ pg/mL	0.82 pg/mL	[158]
	PDA/Fe_3_O_4_	SDS/acetic acid	OVA	10–10^5^ fg/mL	3 fg/mL	[159]
	TMOS-PTEOS/GO	acetic acid	OVA	0.1–10^5^ pg/mL	0.02 pg/mL	[160]
	polypyrrole-PcFe-SA/AuNPs	HCl	S protein	0.1–10^5^ pg/mL	30.1 fg/mL	[161]
	p(L-Cys)/AuNPs/Co, Mo_2_C-CNF	acetic acid–acetonitrile	HRP	0.01–10^5^ pg/mL	7.4 fg/mL	[162]
	polypyrrole/NH_2_-G/AuNBs	HCl	anti-IgG	50–10^5^ pg/mL	17 pg/mL	[163]
	PDA/pMB/ANME	PBS/methanol	OPN	10–10^6^ pg/mL	3 pg/mL	[164]
	VPBA-MA/AuNPs-GO	SDS/HCl	HRP	1–10^5^ ng/mL	0.57 pg/mL	[165]
	Fe_3_O_4_@Au nanofibers	SDS/sulfuric acid	HRP	10–300 μg/mL	5 μg/mL	[166]
	TMOS−PTEOS/Au	acetic acid	OVA	1–10^6^ pg/mL	0.87 pg/mL	[167]

Abbreviation: PEDOT, poly(3,4-ethylenedioxythiophene); LSG, laser scribed graphene; AuNS nanostructured gold; PAP, poly(o-aminophenol); VBTC, vinylbenzyl trimethylammonium chloride; CWP, conductive wax-paper; APTES, 3-aminopropyl triethoxysilane; TEOS, tetraethoxysilane; PVC, poly(vinyl chloride); GO, graphene oxide; PTB, poly(toluidine blue); mPD, meta-phenylenediamine; AMPTMA, (3-acrylamidopropyl)trimethylammonium chloride; poly-oPD, poly(o-phenylenediamine); Cu-MOF, Cu-based metal–organic framework; EG-FET, extended-gate field-effect transistors; IL, ionic liquid; AuNPs, gold nanoparticles; rGO, reduced graphene oxide; TFME, thin film metal electrodes; PAPBA, poly(3-aminophenylboronic acid); 3-TBA, 3-thiophene boric acid; pTH, poly(thionine); pABSA, poly(p-aminobenzene sulfonic acid); PDA, polydopamine; TMOS-PTEOS, tetramethoxysilane-phenyltriethoxysilane; PcFe, ferrous phthalocyanine; SA, sodium alginate; CNF, carbon nanofibers; NH_2_-G, aminated graphene; AuNBs, gold nanobipyramids; pMB, poly(methylene blue); ANME, acupuncture needle microelectrode; VPBA, 4-vinylphenylboronic acid; MA, methacrylic acid; MEth, 2-mercaptoethanol; DTT, dithiothreitol; Her-2, human epidermal growth factor receptor 2; Myo, myoglobin; CA 15-3, cancer antigen 15-3; BSA, bovine serum albumin; rhEPO, recombinant human erythropoietin; PSA, prostate specific antigen; EGFR, epidermal growth factor receptor; VEGF, vascular endothelial growth factor; OVA, ovalbumin; HSA, human serum albumin; IL-6, interleukin 6; hCG, human chorionic gonadotrophin; CEA, carcinoembryonic antigen; MAA, serum amyloid A; BHb, bovine hemoglobin; BDNF, brain-derived neurotrophic factor; ncovS1, SARS-CoV-2 spike protein subunit S1; HCV E2, hepatitis C virus envelope protein E2; CDNF, cerebral dopamine neurotrophic factor; HRP, horseradish peroxidase; OPN, osteopontin.

Protein immobilization is a critical step in down-top imprinting approaches, as it directly modulates the specificity, bioactivity, and reusability of the MIPs. Each immobilization method exhibits distinct mechanisms, experimental conditions, and inherent advantages and limitations. As shown in Table 4, physical adsorption or entrapment is a straightforward approach, but the harsh polymerization conditions may cause protein denaturation. In addition, template removal is inefficient and tends to damage the polymer structure, resulting in poor sensing performance. Covalent coupling methods including amide bond coupling, glutaraldehyde crosslinking, disulfide bond coupling, and others rely on irreversible or slowly reversible chemical bonds to achieve stable protein immobilization, but they may suffer from random protein orientation, which often induces protein denaturation. Additionally, template elution requires harsh conditions that may inevitably damage the polymer matrix. Boronic acid can specifically target cis-diol moieties in glycoproteins, allowing mild and reversible template elution, yet its application is restricted to glycoproteins and the method is susceptible to interference from other cis-diol-containing molecules.

### 3.3. Receptor-Assisted Immobilization

**Figure 4:**
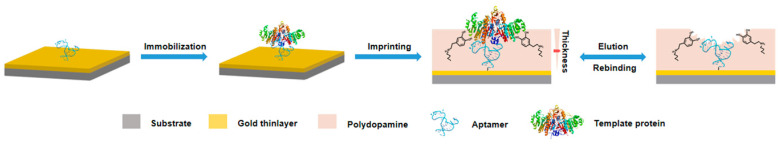
Schematic illustration of the principle and procedure for preparing aptamer−MIP hybrid. Reprinted with permission from ref. [170].

Protein detection technologies can adopt hybrid approaches that combine synthetic or natural receptors with MIPs. To construct molecularly imprinted hybrid systems, receptors with high and specific affinity toward target proteins can be introduced into the backbones. For instance, nucleic acids and other receptors have been used as biorecognition elements to successfully develop dual-recognition strategies, thereby overcoming the limitations of traditional molecular imprinting techniques. The advantages of this strategy include good biocompatibility, simple preparation procedure, high selectivity, and low non-specific binding. Multiple strategies have been developed for protein orientation, such as host-guest interactions and aptamer-assisted immobilization [168,169]. Aptamers are single-stranded oligonucleotides that can fold into well-defined three-dimensional structures. Due to their excellent recognition capability, aptamers have become ideal biorecognition elements for various sensing devices. These oligonucleotides can specifically bind with targets ranging from small molecules to macromolecules such as proteins and even entire cells. However, the affinity of aptamers may be affected by their conformational diversity. Therefore, aptamers can often combine with molecular imprinting to overcome the drawbacks. Although molecular imprinting and aptamers each have some advantages and limitations, studies have demonstrated that integrating aptamers into imprinted cavities can significantly improve the affinity of imprinted materials [170]. In such hybrid aptamer-MIP systems, aptamers are used to immobilize proteins on the surfaces of metal electrodes and nanoparticles (Figure 4). Functional monomers are then polymerized controllably around the aptamer–protein complexes to encapsulate the template, improving the selectivity of MIPs. A recent study reported a facile and rapid functionalization of gold electrodes with PSA, followed by electropolymerization of dopamine to fabricate a hybrid aptamer–MIP architecture [171]. A thiolated DNA aptamer with high affinity toward the target was used as a linking agent. After the elution of PSA, the remaining imprinted sites and high-affinity aptamers on the electrode surface synergistically and greatly enhanced the recognition performance of MIPs. The biosensor exhibited high sensitivity with a detection limit of 1 pg/mL. Such highly ordered topological structure also improved the analytical selectivity of biosensors toward target antigens.

### 3.4. Epitope-Based Imprinting

Efficient molecular imprinting for macromolecules remains challenging due to their large molecular weight, unstable conformation, and complex structure. First, the polymer matrix formed during polymerization is full of pores or cavities corresponding to target molecules. The bulky size of proteins makes it difficult for them to be completely removed from the matrix and thus hinders the formation of effective recognition sites. Second, protein conformation is susceptible to various environmental factors during imprinting such as temperature, pH, and organic solvents. These factors may consequently reduce the binding affinity of the imprinted cavities toward the target proteins. Third, unlike small-molecule templates, structurally complex proteins possess numerous functional groups that readily induce non-specific binding. Such interactions tend to lower the selectivity of MIPs. In addition, the high purity requirement in the synthesis of MIPs leads to relatively high costs when using proteins directly as templates. Although MIPs play a significant role in protein recognition, novel strategies are still required to further improve the sensing performance. Epitopes are small active regions within protein structures that can act as binding sites for antigens or targets. Epitope imprinting is a novel approach, successfully developed by Rachkov and Minoura to prepare MIPs for the separation and analysis of proteins [172]. The core of this strategy lies in the use of short peptides instead of intact proteins as templates for synthesizing molecularly imprinted materials. The peptide templates are usually selected according to the amino acid sequences exposed on the external regions of target proteins, since these regions are most likely to interact with the imprinted films. After the peptides are imprinted into the polymeric films, the binding sites only interact with specific segments of target proteins, which avoids imprinting obstacles caused by the large size and complex structure of whole proteins. Compared with intact proteins, epitopes have predictable primary and/or secondary structures due to their short length, whereas intact proteins possess complicated tertiary structures with ambiguous recognition sites. Such stable and definable structures of epitopes can well meet the requirements of molecular imprinting applications. Imprinting based on protein recognition motifs helps standardize the structure of imprinted cavities and thus more effectively regulate the selectivity of sensors [173].

**Figure 5:**
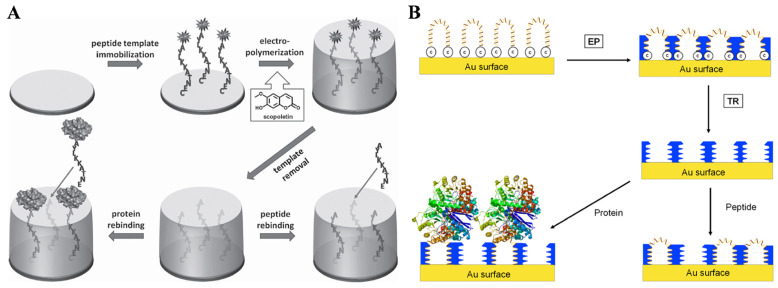
(**A**) Schematic representation of the epitope-oriented surface imprinting approach for preparation of MIP ultrathin films. Reprinted with permission from ref. [174]. (**B**) Generation of SAM epitope bridges on gold surfaces using doubly cysteine-modified peptide templates for subsequent molecular imprinting via electropolymerization and target sensing. Reprinted with permission from ref. [181].

Various approaches are available for the selection of epitope templates, among which the use of C-terminal fragments is much more common, probably due to their better matching with terminal regions of target molecules. The advantages of this strategy include easy and low-cost preparation of linear peptides, rapid screening of suitable binding sites only based on amino acid sequences of proteins, and convenient modification of binding sites by introducing specific amino acids such as histidine and cysteine to facilitate desired bioconjugation reactions. For example, Dechtrirat et al. fabricated an ultra-thin, surface-constrained MIP film by modifying a cysteine-labeled peptide template on the gold surface (Figure 5A) [174]. After electropolymerization of scopoletin, the template was removed by electrochemically. The MIP film can be able to selectively capture the template peptide and target protein.

The commonly used epitope sources for imprinting include peptide sequences derived from target proteins, naturally existing epitopes, nonlinear peptides, and non-peptidic epitopes [175,176,177,178,179,180]. For peptide sequences derived from proteins, structural analysis of target proteins is required to ensure compatibility with synthesis conditions. Natural epitopes employ protein fragments known to be recognized by antibodies or cellular receptors. Nonlinear peptides are designed because natural molecular recognition often relies on interactions with secondary and tertiary structures of proteins, making linear peptides an oversimplified choice. In addition, non-peptidic epitopes including monosaccharides and oligosaccharides can be used to design recognition elements for glycoproteins or detect cells decorated with such molecules. By using a cysteine-included peptide as the template, Drzazgowska et al. developed an epitope imprinting technique based on the formation of SAM bridges on gold electrodes in combination of polymerization to produce a polymer network (Figure 5B) [181]. A 10-mer peptide of neuron specific enolase (NSE) biomarker (LKAVDHINST) was used as the imprinting template. Its modified version (CKGVLKAVDHISTAPC) was used as the template and scopolamine was used as the functional monomer for imprinting. The target protein was maintained in the pore through the size, shape, and hydrogen bonding of surface epitope. With this method, NSE in serum samples was determine with a linear range of 1.25~100 ng/mL and a detection limit of 0.25 M.

**Figure 6:**
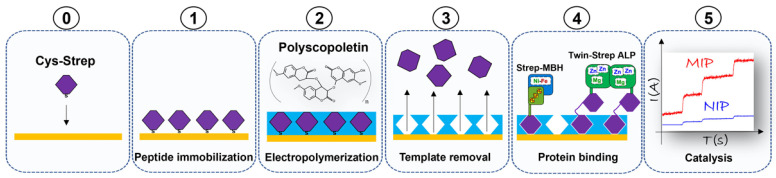
Schematic representation of the workflow utilized for Strep-MIP synthesis using a Strep-tag II peptide extended with a Cys residue at its C-terminus (Cys-Strep) as epitope and scopoletin (SP) as electroactive monomer. (0,1) Cys-Strep chemisorption on a gold surface via the thiolate group; (2) SP electropolymerization around the chemisorbed Cys-Strep; (3) Cys-Strep template removal by anodic polarization; (4) Binding of either Strep-MBH or Twin-Strep-ALP targets; (5) Biocatalytic assays utilizing enzymes immobilized via imprinted (MIP) and non-imprinted (NIP) sites. Reprinted with permission from ref. [178].

Epitope imprinting employing a peptide epitope as the template enables mild polymerization conditions, facile template elution, and high specificity, while its main limitation lies in the relatively low binding affinity for full-length target proteins. A Au–S bond is mostly used for gold-based electrode by forming a stable coordination bond between sulfhydryl group on the surface of protein and gold atom. Template desorption can be realized through competitive substitution by high-concentration sulfhydryl compounds or electrochemical desorption. MIP-based electrochemical biosensors have been developed for the detection of various proteins, such as cytochrome c, alpha-fetoprotein, and the receptor binding domain of SARS-CoV-2 spike protein, in which the templates were removed through electrochemical oxidation and thorough washing [182,183,184,185]. Recently, Yarman et al. developed a Strep-MIP platform for identifying recombinant proteins labeled with an affinity peptide Strep-Tag II (Figure 6) [178]. The peptide with a cysteine residue was anchored on a gold electrode through the formation of a Au–S bond. After electropolymerization of scopoletin, the peptide template was removed by applying an anodic potential. The Strep-MIP platform has been used to immobilize Strep-labeled O_2_-resistant membrane-bound [NiFe]-hydrogenase (Strep-MBH) and bacterial Twin-Strep-tagged alkaline phosphatase (Twin-Stre p-ALP) as the model analytes. The immobilized enzymes maintained excellent catalytic activity even after multiple washing cycles. This work provides a new approach for the preparation of MIPs to recognize and detect proteins.

### 3.5. Imprinted SAMs

**Figure 7:**
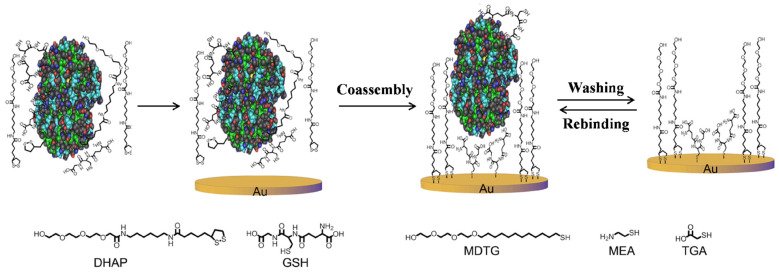
Schematic representation of preparation of a protein-imprinted SAM on a gold electrode and chemical structures of relevant thiols. Reprinted with permission from ref. [187].

In order to improve the binding selectivity and stability of imprinting cavities, monomers can be functionalized with amino (NH^3+^), carboxyl (COO^−^), hydroxyl (OH), benzyl (C_6_H_6_), and propyl (CH_2_CH_2_CH_3_) groups to promote ionic and hydrogen bonds as well as hydrophobic interactions [186]. However, special attention should be paid to optimizing the monomer ratio and polymerization time when using multiple monomers for the preparation of MIPs. SAMs with imprinted patterns have been considered promising alternatives to typical MIPs since they can avoid the use of polymerization initiators and organic solvents and favor the remove and rebinding of template proteins. In addition, the thickness of imprinted SAMs can be precisely controlled by the length of polymer monomers. For example, thiolated poly(ethylene glycol) (PEG) can be assembled onto protein-anchored gold surfaces, providing recognition sites by the formation of imprinting cavities and enhancing the binding stability through hydrophobic and hydrogen-bonding interactions [187,188]. Zhang et al. found that protein-imprinted SAMs, created with multiple binding sites and biocompatible imprinted cavities from functional thiols and disulfide compounds containing an oligoethylene glycol (OEG) terminal moiety and two amide groups incorporated in the chain (DHAP), showed highly specific binding with target proteins (Figure 7) [187]. With BHb as a mode analyte, the electrochemical signal decreased with the increase in target concentration up to about 100 μg/mL. The work opens the possibility of controlled assembly of intellectual biomaterials and the design of novel electrochemical biosensors.

## 4. Conclusions and Future Perspectives

In summary, molecular imprinting provides a feasible and effective strategy for the recognition and separation of biomacromolecules such as proteins, demonstrating broad application prospects in the fields of sensing and analysis. The rational construction of imprinting cavities, effective removal of templates, reduction of mass transfer resistance, and inhibition of non-specific adsorption are the key issues determining the performance of molecular imprinting materials. At present, the selection of functional monomers still mainly relies on empirical attempts, and its systematic screening mechanism and theoretical basis need to be improved. In addition, the types of proteins used for the preparation of MIPs are very limited, mainly referring to ordinary inexpensive proteins such as BSA and OVA that have favorable characteristics for the imprinting process. Real-sample validation and operational stability were not systematically and comprehensively investigated in most of the reported works, leading to the lack of standardized procedures for the preparation of imprinted sensor electrodes. Proteins with good conformational stability and special physicochemical properties and functions are more favored. Furthermore, the non-specific adsorption in complex biological systems and insufficient stability in recognizing low abundance targets seriously restrict the practical application of molecular imprinting biosensors, especially for the detection of cancer biomarkers. From these aspects, imprinting techniques used for electrochemical detection of protein biomarkers are still in the conceptual validation stage and further improvements are desired in terms of high affinity and excellent selectivity as well as stability. Overall, although there are currently numerous studies dedicated to developing MIP-based electrochemical biosensors based on nanomaterials, conductive polymers, and bioreactors, there are still many challenges in detecting low concentration proteins.

Future research directions can be summarized as follows: (1) developing novel and effective imprinting strategies, such as multi-point collaborative recognition, reversible covalent imprinting, boronic acid-assisted imprinting, and biomimetic imprinting, to improve the binding selectivity and affinity for protein biomarkers; (2) establishing a scientifically universal principle for selecting functional monomers based on target structure, functional groups, hydrophilicity/hydrophobicity, and charge properties, replacing traditional empirical screening; (3) strengthening the anti-fouling modification of imprinting interfaces and developing mild template removal methods to effectively reduce non-specific adsorption and maintain the integrity and stability of imprinted cavities; (4) introducing machine learning and computational simulation to intelligently optimize polymerization conditions, monomer ratios, reaction parameters, and template removal schemes, achieving high-precision prediction and design of imprinting materials. With the continuous development of the above strategies, molecular imprinting will further demonstrate superior performance in the specific recognition and sensitive detection of protein biomarkers, providing a reliable technical platform for clinical detection and early diagnosis.

## Figures and Tables

**Table 2:** Performance of electrochemical biosensors with MIPs as receptors prepared in situ through top-down approaches for protein detection.

MIP Materials	Potential	Elution Reagent	Protein	Linear Range	Detection Limit	Ref.
Polyphenol/AuSPE	−0.2~1.0 V	acetic acid/SDS	HER2-ECD	10–70 ng/mL	1.6 pg/mL	[84]
Polyphenol/AuNE	0~0.9 V	1% acetic acid and 3% SDS	CA 125	0.5–400 U/mL	0.5 U/mL	[85]
Polyphenol/SWCNT	−0.2~0.8 V	proteinase K	FFPM	0.01–60 g/mL	0.6 ng/mL	[86]
PolyAMP	−0.2~1.2 V	proteinase K	Tau	2.18–2.18 × 10^3^ pM	0.02 pM	[87]
PolyAMP	−0.2~0.8 V	0.5 M oxalic acid	galectin-3	0.5–5 × 10^3^ ng/mL	0.5 ng/mL	[88]
PolyAMP/SWCNT	−0.2~0.8 V	proteinase K	BSA	23.8–4.76 × 10^12^ pM	16.83 nM	[89]
PDA/MB@PDMS-S/P-GO	−0.6~0.8 V	Not reported	BSA	1–10^8^ fg/mL	389 pg/mL	[90]
PDA/PEG/GDY	−	acetone	CRP	0.1–10^3^ fg/mL	4.1 fg/mL	[91]
PDA/rGO	−	1 M HCl + 0.01 g/mL SDS	GFAP	1–10^6^ fg mL	754.5 ag/mL	[92]
poly-oPD	0~0.8 V	0.1 M NaOH	BuChE	0.05–2 nM	14.7 pM	[93]
poly-oPD	0~1.1 V	0.1 M H_2_SO_4_	cyt c	0.1–10^3^ pM	3.4 fM	[94]
poly-oPD/CI-HME	−0.4~0.8 V	trypsin + oxalic acid	Aβ42	0.1–10^3^ ng/mL	0.1 ng/mL	[95]
Poly-oPD/QDs	−0.4~1.0 V	acetonitrile/acetic acid (95:5 *v*/*v*)	BKV VP1	0.1–10^5^ fg/mL	0.51 pg/mL	[96]
poly-oPD/P(C2R)	0~1.1 V	0.1 M NaOH	IL-1β	0.1–1.05 × 10^12^ pg/mL	0.23 pg/mL	[97]
poly-oPD/NMIs/rGO	0~0.8 V	0.1 M NaOH + ethanol (4:1 *v*/*v*)	H-FABP	1−10^8^ fg/mL	2.29 fg/mL	[98]
poly-oPD/polypyrrole/AuNCs	−0.2~1.2 V	Not reported	PD-L1	0.1–10^3^ ng/mL	71 pg/mL	[99]
poly-oPD/prussian blue	−0.2~0.6 V	0.1 M HCl or 0.5 M acetic acid	TnI	10–100 pg/mL	3.6 pg/mL	[100]
poly-oPD/ZCISeP QDs	−0.4~1.0 V	1:1 *v*/*v* ethanol, 0.25 mM NaOH, 135 mM NaCl, 1% SDS, 0.1% Tween20 solution	S1 protein	1−10^5^ fg/mL	0.34 pg/mL	[101]
Poly(scopoletin)	0~0.9 V	−0.2 to 1.0 V	ferritin	0–0.5 μM	97 nM	[102]
Poly(scopoletin)/SAM	0~0.9 V	1 M H_2_SO_4_	cyt c	0–4 μM	−	[103]
Poly(scopoletin)	0~1.0 V	NaOH/SDS, NaOH/Tween20	ferritin	0.037–3.36 μM	10 nM	[104]
Polyaniline/chitosan	−0.2~1.2 V	methanol-acetic acid	CRP	1–10^3^ pg/mL	2.22 pg/mL	[105]
Polyaniline/FTO glass	0.2~0.9 V	proteinase K	CEA	0.013–1.75 ng/mL	0.025 ng/mL	[106]
Polyaniline nanotubes	−	1 M HCl	HRP	1–10^10^ pg/mL	0.356 ng/mL	[107]
Poly(α-CD)/polyaniline	−0.2~1.3 V	0.1 M oxalic acid	Aβ42	0.25−8.75 ng/mL	0.20 ng/mL	[108]
Polypyrrole	0~0.95 V	0.2% (*w*/*v*) SDS	S protein	50–125 fg/mL	6.8 fg/mL	[111]
Polypyrrole	1.05 V	0.05 M H_2_SO_4_	S protein	0–25 μg/mL	−	[112]
Polypyrrole	−0.2~1.0 V	saturated NaCl	PSA	0.03–3 × 10^8^ fg/mL	0.03 fg/mL	[113]
Polypyrrole	0~0.9 V	1 NaOH	insulin	20–70 pM	1.9 pM	[114]
Polypyrrole	−0.6~1.6 V	NaOH/ethanol	DJ-1	1–500 nM	1 nM	[115]
Polypyrrole	−0.2~1.2 V	5 mM oxalic acid	PSA	0.01–4.0 ng/mL	2 pg/mL	[116]
Polypyrrole	0.1~1.2 V	25 mM SDS + 10% methanol	Cor a 14	0.1–10^9^ pg/mL	24.5 fg/mL	[117]
Polypyrrole/SAM	−0.3~0.8 V	0.1 M H_2_SO_4_	rN	0–35 nM	0.4 nM	[118]
Polypyrrole/Chitosan	0~0.8 V	0.5 M acetic acid	BSA	0.1–100 pg/mL	0.05 pg/mL	[119]
Polypyrrole/NCD-G	0~0.9 V	5 mM oxalic acid	Aβ42	5–70 pg/mL	1 pg/mL	[120]
Polypyrrole/CNT-PAH/Pt	−0.8~1.1 V	trypsin	STEAP1	0.13–1.3 × 10^10^ fg/mL	−	[121]
Polypyrrole/CS/MWCNT	−0.4~0.6 V	1 M H_2_SO_4_	BSA	0.1–5 × 10^4^ pg/mL	45 fg/mL	[122]
Polypyrrole/AuNS	0~0.9 V	0.05 M H_2_SO_4_ + 5 mM SDS	rN	0–10 ng/mL	51.2 pg/mL	[123]
Polypyrrole/MXene/MWCNT	0.2~1.0 V	1 M NaCl	Aβ_42_	1−100 fg/mL	0.3 fg/mL	[124]
Polypyrrole/IL/graphene	−0.2~1.2 V	1 M H_2_SO_4_	BHb	0.1–10^6^ pg/mL	30.9 fg/mL	[125]
Polypyrrole/MNP/MWNT	0.3~0.8 V	5% acetic acid + 10% SDS	BSA	0.1–10^5^ ng/mL	2.8 fg/mL	[126]

Abbreviation: AuSPE, gold screen-printed electrodes; AuNE, gold nanoelectrode; SWCNT, single-walled carbon nanotube; AMP, aminophenol; PDA, polydopamine; MB, methylene blue; PDMS-S/P-GO, polydimethylsiloxane precursor onto sulfur and phosphorus-doped graphene oxide; GDY, graphdiyne; rGO, reduced graphene oxide; poly-oPD, poly(o-phenylenediamine); CI-HME, carbon ink homemade electrode; QDs, quantum dots; NMIs, gold nano/micro-islands; AuNCs, gold nanoclusters; P(C2R), poly(chromotrope 2R); SAM, self-assembled monolayer; poly(α-CD), poly(α-cyclodextrin); PANTs, polyaniline nanotubes; NCD-G, nitrogen-doped carbon-dot-graphene nanohybrid; MWCNT-PAH/Pt, platinum nanoparticles supported on multiwalled carbon nanotube and functionalized with polyallylamine; CS, chitosan; AuNS, gold nanostructures; MNP, magnetic nanoparticles; HER2-ECD, extracellular domain of human epidermal growth factor receptor 2; CA 125, cancer antigen-125; FFPM, flagellar filaments from Proteus mirabilis; BSA, bovine serum albumin; CRP, C-reactive protein; GFAP, glial fibrillary acidic protein; BuChE, butyrylcholinesterase; cyt c, cytochrome c; Aβ42, an amyloid-β peptide; BKV, BK polyomavirus; IL-1β, cytokine interleukin-1β; H-FABP, heat-fatty acid binding protein; PD-L1, programmed cell death ligand 1; TnI, Troponin I; CEA, carcinoembryonic antigen; HRP, horseradish peroxidase; PSA, prostate specific antigen; DJ-1, a protein deglycase; Cor a 14, Cor a 14-allergen; rN, nucleocapsid protein; STEAP1, six-transmembrane epithelial antigen of the prostate 1; BHb, bovine hemoglobin.

**Table 4:** Comparison of different immobilization methods for top-down protein imprinting.

Immobilization Method	Template Specificity	Protein Orientation	Protein Conformation	Template Removal
Physical adsorption	Low/depending on three-dimensional configuration and non-specific interactions	Random/depending on polymerization process	Prone to denaturation; harsh polymerization conditions can alternative the structures	Very difficult; requiring mechanical grinding or harsh solvent extraction that may lead to severe template leakage and polymer damage
Amide bond	None/coupling with any primary amine-containing molecules	Random/depending on the distribution of surface-accessible amine residues	Potentially altered; covalent reaction may disrupt the local microenvironment and biological activity	Difficult; requiring harsh denaturing conditions that easily damage the imprinted polymer layer
Disulfide bond	Moderate/coupling with proteins containing surface-exposed free thiols	Semi-oriented/depending on the accessibility of thiols	Potentially altered; coupling depends on the location of thiols in the proteins	Reversible via reducing agents, but may compromise the long-term stability of imprinted layer
Schiff-base	None/coupling with any primary amine-containing molecules	Completely random/depending on the crosslinking uncontrollable sites	Severe disruption; vigorous crosslinking often leads to irreversible conformational fixation or denaturation	Extremely difficult; requiring the use of strong acid/alkali or protease that may damage the imprinted layer
Boronic acid	High but prone to interference from other cis-diol-containing molecules	Semi-oriented/depending on the position of glycosylation sites	Potentially affected; binding may induce local conformational changes in sugar chain moieties	Mild and reversible; requiring the use of weakly acidic solutions or competing eluents

## Data Availability

No new data were created or analyzed in this study.

## References

[1] Guo L., Zhao Y., Huang Q., Huang J., Tao Y., Chen J., Li H.-Y., Liu H. (2024). Electrochemical protein biosensors for disease marker detection: Progress and opportunities. Microsyst. Nanoeng..

[2] López Mujica M.E.J., Ferapontova E.E. (2026). Electrochemical biosensors for cancer diagnosis and prognosis using protein biomarkers: Current trends, advances, and clinical translation potential. Sensors.

[3] Mostafa A.M., Barton S.J., Wren S.P., Barker J. (2021). Review on molecularly imprinted polymers with a focus on their application to the analysis of protein biomarkers. TrAC-Trend. Anal. Chem..

[4] Selvolini G., Marrazza G. (2017). MIP-based sensors: Promising new tools for cancer biomarker determination. Sensors.

[5] Frasco M., Truta L., Sales M., Moreira F. (2017). Imprinting technology in electrochemical biomimetic sensors. Sensors.

[6] Khan A., Anwar M., Rehman A.U., Shokouhimehr M., Reis N.M., Kalhoro K.A., Zhang C., Liu Z. (2025). Biorecognition-based electrochemical sensors for highly sensitive C-reactive protein detection: A review. Int. J. Biol. Macromol..

[7] Shah N.S., Thotathil V., Zaidi S.A., Sheikh H., Mohamed M., Qureshi A., Sadasivuni K.K. (2022). Picomolar or beyond limit of detection using molecularly imprinted polymer-based electrochemical sensors: A review. Biosensors.

[8] Liu L., Liu J., Zhang W., Liang Z., Zhang Y., Liu J., Yang K., Zhang L. (2025). Bio-imprinted materials towards artificial antibodies: Technique, application and perspective. TrAC-Trend. Anal. Chem..

[9] Akgönüllü S., Kılıç S., Esen C., Denizli A. (2023). Molecularly imprinted polymer-based sensors for protein detection. Polymers.

[10] Gritsok D., Hedström M., Montenegro M.C.B.S.M., Amorim C.G. (2025). Electrochemical molecularly imprinted polymer sensors in viral diagnostics: Innovations, challenges and case studies. Biosens. Bioelectron..

[11] Sarvutiene J., Prentice U., Ramanavicius S., Ramanavicius A. (2024). Molecular imprinting technology for biomedical applications. Biotechnol. Adv..

[12] Yarman A., Kurbanoglu S., Zebger I., Scheller F.W. (2021). Simple and robust: The claims of protein sensing by molecularly imprinted polymers. Sens. Actuators B Chem..

[13] Zidarič T., Finšgar M., Maver U., Maver T. (2022). Artificial biomimetic electrochemical assemblies. Biosensors.

[14] Ansari S., Masoum S. (2019). Molecularly imprinted polymers for capturing and sensing proteins: Current progress and future implications. TrAC-Trend. Anal. Chem..

[15] Erdem A., Senturk H., Karakus M. (2025). Molecularly imprinted polymer-based sensors: Design and advances in the analysis of DNA and protein. Talanta Open.

[16] Krishnan H., Gopinath S.C.B. (2025). Current applications and considerations of macromolecular imprinted polymer on electrochemical transducing sensing surfaces in clinical diagnosis. Microchem. J..

[17] Saylan Y., Yilmaz F., Özgür E., Derazshamshir A., Yavuz H., Denizli A. (2017). Molecular imprinting of macromolecules for sensor applications. Sensors.

[18] Chen G., Zhang S., Ma X., Wilson G., Zong R., Fu Q. (2024). Antibody mimics for precise identification of proteins based on molecularly imprinted polymers: Developments and prospects. Chem. Eng. J..

[19] Resina L., Alemán C., Ferreira F.C., Esteves T. (2023). Protein-imprinted polymers: How far have “plastic antibodies” come?. Biotechnol. Adv..

[20] Li Y., Luo L., Kong Y., Li Y., Wang Q., Wang M., Li Y., Davenport A., Li B. (2024). Recent advances in molecularly imprinted polymer-based electrochemical sensors. Biosens. Bioelectron..

[21] Gui R., Jin H., Guo H., Wang Z. (2018). Recent advances and future prospects in molecularly imprinted polymers-based electrochemical biosensors. Biosens. Bioelectron..

[22] Ben Moussa F. (2023). Molecularly imprinted polymers meet electrochemical cancer chemosensors: A critical review from a clinical and economic perspective. Microchem. J..

[23] Mazzotta E., Di Giulio T., Malitesta C. (2022). Electrochemical sensing of macromolecules based on molecularly imprinted polymers: Challenges, successful strategies, and opportunities. Anal. Bioanal. Chem..

[24] Silva A.T., Figueiredo R., Azenha M., Jorge P.A.S., Pereira C.M., Ribeiro J.A. (2023). Imprinted hydrogel nanoparticles for protein biosensing: A review. ACS Sens..

[25] Erdőssy J., Horváth V., Yarman A., Scheller F.W., Gyurcsányi R.E. (2016). Electrosynthesized molecularly imprinted polymers for protein recognition. TrAC-Trend. Anal. Chem..

[26] Zhang Y., Zhang H., Shi L. (2026). Progress in the preparation of advanced molecularly imprinted polymers via surface template-anchoring methods. TrAC-Trend. Anal. Chem..

[27] Qu K., Li J. (2024). Functional interface for glycoprotein sensing: Focusing on biosensors. Langmuir.

[28] Li P., Liu Z. (2024). Glycan-specific molecularly imprinted polymers towards cancer diagnostics: Merits, applications, and future perspectives. Chem. Soc. Rev..

[29] Ali M.M., Liu X., Amin F.R., Zhou J., Hu L. (2025). Recent advances in molecularly imprinted polymers for glycosylated molecule analysis. TrAC-Trend. Anal. Chem..

[30] Dietl S., Sobek H., Mizaikoff B. (2021). Epitope-imprinted polymers for biomacromolecules: Recent strategies, future challenges and selected applications. TrAC-Trend. Anal. Chem..

[31] Wang X., Chen G., Zhang P., Jia Q. (2021). Advances in epitope molecularly imprinted polymers for protein detection: A review. Anal. Methods.

[32] Brazys E., Ratautaite V., Mohsenzadeh E., Boguzaite R., Ramanaviciute A., Ramanavicius A. (2025). Formation of molecularly imprinted polymers: Strategies applied for the removal of protein template (review). Adv. Colloid Interf. Sci..

[33] Boroznjak R., Reut J., Tretjakov A., Lomaka A., Öpik A., Syritski V. (2017). A computational approach to study functional monomer-protein molecular interactions to optimize protein molecular imprinting. J. Mol. Recogn..

[34] Mofazali P., Lalinia M., Gross J.D., Samadi A. (2026). Molecularly imprinted polymers: Applications, computational approaches, and the transformative role of artificial intelligence. Coord. Chem. Rev..

[35] Rajpal S., Mizaikoff B. (2022). An in silico predictive method to select multi-monomer combinations for peptide imprinting. J. Mater. Chem. B.

[36] Dashtaki R.M., Dashtaki S.M., Heydari-Bafrooei E., Piran M.J. (2025). Enhancing the predictive performance of molecularly imprinted polymer-based electrochemical sensors using a stacking regressor ensemble of machine learning models. ACS Sens..

[37] Mohsenzadeh E., Ratautaite V., Brazys E., Ramanavicius S., Zukauskas S., Plausinaitis D., Ramanavicius A. (2024). Design of molecularly imprinted polymers (MIP) using computational methods: A review of strategies and approaches. WIREs Comput. Mol. Sci..

[38] Ann Maria C.G., Varghese A., Nidhin M. (2021). Recent advances in nanomaterials based molecularly imprinted electrochemical sensors. Crit. Rev. Anal. Chem..

[39] Pan M., Hong L., Xie X., Liu K., Yang J., Wang S. (2020). Nanomaterials-based surface protein imprinted polymers: Synthesis and medical applications. Macromol. Chem. Phys..

[40] Dong X., Zhang C., Du X., Zhang Z. (2022). Recent advances of nanomaterials-based molecularly imprinted electrochemical sensors. Nanomaterials.

[41] Singh R., Singh M. (2025). Perspective towards nanomaterial-integrated molecularly imprinted polymer (MIP)-based electrochemical sensors for protein biomarkers detection: A review. Microchem. J..

[42] Somnet K., Wanram S., Chairam S., Jarujamrus P., Nacapricha D., Lieberzeit P.A., Amatatongchai M. (2025). Ultrasensitive and selective impedance paper-based analytical device through Dual-C imprinted sensor for determination of carcinoembryonic antigen and C-reactive protein. Microchim. Acta.

[43] Zhao W., Li B., Xu S., Zhu Y., Liu X. (2020). A fabrication strategy for protein sensors based on an electroactive molecularly imprinted polymer: Cases of bovine serum albumin and trypsin sensing. Anal. Chim. Acta.

[44] Zhao W., Li B., Xu S., Huang X., Luo J., Zhu Y., Liu X. (2019). Electrochemical protein recognition based on macromolecular self-assembly of molecularly imprinted polymer: A new strategy to mimic antibody for label-free biosensing. J. Mater. Chem. B.

[45] Liang A., Tang B., Hou H., Sun L., Luo A. (2019). A novel CuFe_2_O_4_ nanospheres molecularly imprinted polymers modified electrochemical sensor for lysozyme determination. J. Electroanal. Chem..

[46] Luo J., Jiang S., Liu X. (2014). Electrochemical sensor for bovine hemoglobin based on a novel graphene-molecular imprinted polymers composite as recognition element. Sens. Actuators B Chem..

[47] Luo J., Huang J., Wu Y., Sun J., Wei W., Liu X. (2017). Synthesis of hydrophilic and conductive molecularly imprinted polyaniline particles for the sensitive and selective protein detection. Biosens. Bioelectron..

[48] Sun B., Ni X., Cao Y., Cao G. (2017). Electrochemical sensor based on magnetic molecularly imprinted nanoparticles modified magnetic electrode for determination of Hb. Biosens. Bioelectron..

[49] Mandani S., Rezaei B., Ensafi A.A., Rezaei P. (2021). Ultrasensitive electrochemical molecularly imprinted sensor based on AuE/Ag-MOF@MC for determination of hemoglobin using response surface methodology. Anal. Bioanal. Chem..

[50] Garcia-Cruz A., Ahmad O.S., Alanazi K., Piletska E., Piletsky S.A. (2020). Generic sensor platform based on electro-responsive molecularly imprinted polymer nanoparticles (e-NanoMIPs). Microsyst. Nanoeng..

[51] Kaur S., Singla P., Dann A.J., McClements J., Sullivan M.V., Kim M., Stoufer S., Dawson J.A., Crapnell R.D., Banks C.E. (2024). Sensitive electrochemical and thermal detection of human noroviruses using molecularly imprinted polymer nanoparticles generated against a viral target. ACS Appl. Mater. Interfaces.

[52] McClements J., Bar L., Singla P., Canfarotta F., Thomson A., Czulak J., Johnson R.E., Crapnell R.D., Banks C.E., Payne B. (2022). Molecularly imprinted polymer nanoparticles enable rapid, reliable, and robust point-of-care thermal detection of SARS-CoV-2. ACS Sens..

[53] Canfarotta F., Czulak J., Guerreiro A., Cruz A.G., Piletsky S., Bergdahl G.E., Hedström M., Mattiasson B. (2018). A novel capacitive sensor based on molecularly imprinted nanoparticles as recognition elements. Biosens. Bioelectron..

[54] McClements J., Seumo Tchekwagep P.M., Vilela Strapazon A.L., Canfarotta F., Thomson A., Czulak J., Johnson R.E., Novakovic K., Losada-Pérez P., Zaman A. (2021). Immobilization of molecularly imprinted polymer nanoparticles onto surfaces using different strategies: Evaluating the influence of the functionalized interface on the performance of a thermal assay for the detection of the cardiac biomarker Troponin I. ACS Appl. Mater. Interfaces.

[55] Stephen A.N., Holden M.A., Sullivan M.V., Turner N.W., Dennison S.R., Reddy S.M. (2025). Optimised solution-phase synthesis of nanoMIPs for protein detection in electrochemical diagnostics. Biomed. Mater..

[56] Betlem K., Canfarotta F., Raumbault R., Banks C.E., Eersels K., van Grinsven B., Cleij T.J., Crapnell R., Hudson A., Peeters M. (2020). Thermistors coated with molecularly imprinted nanoparticles for the electrical detection of peptides and proteins. Analyst.

[57] Sun Y., Zhang G., Liu J., Li Y. (2023). Biomimetic sandwich assay of proteins with a photoelectrochemical sensor using molecularly imprinted targeting and self-ratiometric signaling. Sens. Actuators B Chem..

[58] Sun Y., Li S., Yang Y., Feng X., Wang W., Liu Y., Zhao M., Zhang Z. (2019). Fabrication of a thermal responsive hemoglobin (Hb) biosensor via Hb-catalyzed eATRP on the surface of ZnO nanoflowers. J. Electroanal. Chem..

[59] Cui M., Sun X., Liu R., Du M., Song X., Wang S., Hu W., Luo X. (2022). A dual-responsive electrochemical biosensor based on artificial protein imprinted polymers and natural hyaluronic acid for sensitive recognition towards biomarker CD44. Sens. Actuators B Chem..

[60] Wang S., Che Z., Guo C., Liu Y., Yang S., Zhou M., Gong Y., Li T., Cui M., Luo X. (2021). A durable antifouling protein molecularly imprinted gel interface for human serum albumin detection and antibacterial application. Chem. Eng. J..

[61] Lin Y., Guo Z., Dong S., Li Y., Kan B., Zhao K., Li S., Yang Z. (2025). Protein imprinted CaAlg/CaSiO_3_ hybrid hydrogel modified electrochemical sensor for sensitive detection of BSA. Colloid. Surf. A Physicochem. Eng. Asp..

[62] Yang C., Ji X.-F., Cao W.-Q., Wang J., Zhang Q., Zhong T.-L., Wang Y. (2019). An ultra sensitive and selective impedance sensor based on protein-imprinted polymer. Sens. Actuators B Chem..

[63] Yang C., Ji X.-F., Cao W.-Q., Wang J., Zhang Q., Zhong T.-L., Wang Y. (2019). Molecularly imprinted polymer based sensor directly responsive to attomole bovine serum albumin. Talanta.

[64] Wei Y., Zeng Q., Huang J., Guo X., Wang L., Wang L. (2020). Preparation of gas-responsive imprinting hydrogel and their gas-driven switchable affinity for target protein recognition. ACS Appl. Mater. Interfaces.

[65] Wei Y., Yu F., Diao Z., Xu R., Li H., Qin G., Guo X. (2022). Self-cleaning electrochemical protein-imprinting biosensor with a dual-driven switchable affinity for sensing bovine serum albumin. Talanta.

[66] Ding S., Lyu Z., Niu X., Zhou Y., Liu D., Falahati M., Du D., Lin Y. (2020). Integrating ionic liquids with molecular imprinting technology for biorecognition and biosensing: A review. Biosens. Bioelectron..

[67] Jalili F., Jalalvand A.R. (2023). A novel and intelligent molecularly imprinted enzymatic biosensor for biosensing of human serum albumin in the presence of gamma-globulin, and glucose as uncalibrated interference. Sens. Bio-Sens. Res..

[68] Wang Y., Han M., Liu G., Hou X., Huang Y., Wu K., Li C. (2015). Molecularly imprinted electrochemical sensing interface based on in-situ-polymerization of amino-functionalized ionic liquid for specific recognition of bovine serum albumin. Biosens. Bioelectron..

[69] Yang X., Yin Z.-Z., Zheng G., Zhou M., Zhang H., Li J., Cai W., Kong Y. (2023). Molecularly imprinted miniature electrochemical biosensor for SARS-CoV-2 spike protein based on Au nanoparticles and reduced graphene oxide modified acupuncture needle. Bioelectrochemistry.

[70] Wang Y., Han M., Ye X., Wu K., Wu T., Li C. (2017). Voltammetric myoglobin sensor based on a glassy carbon electrode modified with a composite film consisting of carbon nanotubes and a molecularly imprinted polymerized ionic liquid. Microchim. Acta.

[71] Wang C., Ye X., Wang Z., Wu T., Wang Y., Li C. (2017). Molecularly imprinted photo-electrochemical sensor for human epididymis protein 4 based on polymerized ionic liquid hydrogel and gold nanoparticle/ZnCdHgSe quantum dots composite film. Anal. Chem..

[72] Wu Y., Wang Y., Wang X., Wang C., Li C., Wang Z. (2019). Electrochemical sensing of α-fetoprotein based on molecularly imprinted polymerized ionic liquid film on a gold nanoparticle modified electrode surface. Sensors.

[73] Stephen A.N., Dennison S.R., Holden M.A., Reddy S.M. (2023). Rapid sub-nanomolar protein determination in serum using electropolymerized molecularly imprinted polymers (E-MIPs). Analyst.

[74] Balayan S., Chauhan N., Chandra R., Jain U. (2022). Molecular imprinting based electrochemical biosensor for identification of serum amyloid A (SAA), a neonatal sepsis biomarker. Int. J. Biol. Macromol..

[75] Bueno L., El-Sharif H.F., Salles M.O., Boehm R.D., Narayan R.J., Paixão T.R.L.C., Reddy S.M. (2014). MIP-based electrochemical protein profiling. Sens. Actuators B Chem..

[76] Sun Y., Du H., Lan Y., Wang W., Liang Y., Feng C., Yang M. (2016). Preparation of hemoglobin (Hb) imprinted polymer by Hb catalyzed eATRP and its application in biosensor. Biosens. Bioelectron..

[77] Wei Y., Zeng Q., Hu Q., Wang M., Tao J., Wang L. (2018). Self-cleaned electrochemical protein imprinting biosensor basing on a thermo-responsive memory hydrogel. Biosens. Bioelectron..

[78] Balayan S., Chauhan N., Hooda V., Chandra R., Rosario W., Jain U. (2024). Advancing electrochemical sensing: A smart platform for accurate CRP level detection in neonatal septicaemia. Talanta Open.

[79] Iqbal T., Fatima L., Sattar J., Fatima B., Ahmad J., Hina M., Najam ul Haq M., Majeed S. (2024). Molecularly imprinted polymer Zeolitic Imidazolate Framework-8 based electrochemical sensor for BSA detection and its practical applicability. J. Electroanal. Chem..

[80] Kumar D., Prasad B.B. (2012). Multiwalled carbon nanotubes embedded molecularly imprinted polymer-modified screen printed carbon electrode for the quantitative analysis of C-reactive protein. Sens. Actuators B Chem..

[81] Prasad B.B., Prasad A., Tiwari M.P. (2013). Multiwalled carbon nanotubes-ceramic electrode modified with substrate-selective imprinted polymer for ultra-trace detection of bovine serum albumin. Biosens. Bioelectron..

[82] Zhang T., Sun L., Zhang Y. (2021). Highly sensitive electrochemical determination of the SARS-COV-2 antigen based on a gold/graphene imprinted poly-arginine sensor. Anal. Methods.

[83] Duan D., Yang H., Ding Y., Ye D., Li L., Ma G. (2018). Three-dimensional molecularly imprinted electrochemical sensor based on Au NPs@Ti-based metal-organic frameworks for ultra-trace detection of bovine serum albumin. Electrochim. Acta.

[84] Pacheco J.G., Rebelo P., Freitas M., Nouws H.P.A., Delerue-Matos C. (2018). Breast cancer biomarker (HER2-ECD) detection using a molecularly imprinted electrochemical sensor. Sens. Actuators B Chem..

[85] Viswanathan S., Rani C., Ribeiro S., Delerue-Matos C. (2012). Molecular imprinted nanoelectrodes for ultra sensitive detection of ovarian cancer marker. Biosens. Bioelectron..

[86] Khan M.A.R., Aires Cardoso A.R., Sales M.G.F., Merino S., Tomás J.M., Rius F.X., Riu J. (2017). Artificial receptors for the electrochemical detection of bacterial flagellar filaments from Proteus mirabilis. Sens. Actuators B Chem..

[87] Ben Hassine A., Raouafi N., Moreira F.T.C. (2021). Novel electrochemical molecularly imprinted polymer-based biosensor for Tau protein detection. Chemosensors.

[88] Cerqueira S.M.V., Fernandes R., Moreira F.T.C., Sales M.G.F. (2021). Development of an electrochemical biosensor for Galectin-3 detection in point-of-care. Microchem. J..

[89] Khan M.A.R., Moreira F.T.C., Riu J., Ferreira Sales M.G. (2016). Plastic antibody for the electrochemical detection of bacterial surface proteins. Sens. Actuators B Chem..

[90] Chen Y., Su X., Zhang Y., Wei X., Zhang J., Zhang Z., Deng X., Sun S. (2025). Synergistic integration of self-cleaning interface and molecular imprinting in ratiometric electrochemical biosensor: Toward ultrasensitive BSA monitoring. Biosens. Bioelectron..

[91] Cui M., Che Z., Gong Y., Li T., Hu W., Wang S. (2022). A graphdiyne-based protein molecularly imprinted biosensor for highly sensitive human C-reactive protein detection in human serum. Chem. Eng. J..

[92] Li Y., Luo L., Senicar L., Asrosa R., Kizilates B., Xing K., Torres E., Xu L., Li D., Graham N. (2024). An ultrasensitive molecularly imprinted point-of-care electrochemical sensor for detection of glial fibrillary acidic protein. Adv. Healthc. Mater..

[93] Ozcelikay G., Kurbanoglu S., Zhang X., Kosak Soz C., Wollenberger U., Ozkan S.A., Yarman A., Scheller F.W. (2019). Electrochemical MIP sensor for butyrylcholinesterase. Polymers.

[94] Campagnol D., Karimian N., Paladin D., Rizzolio F., Ugo P. (2022). Molecularly imprinted electrochemical sensor for the ultrasensitive detection of cytochrome c. Bioelectrochemistry.

[95] Pereira M.V., Marques A.C., Oliveira D., Martins R., Moreira F.T.C., Sales M.G.F., Fortunato E. (2020). Paper-based platform with an in situ molecularly imprinted polymer for β-amyloid. ACS Omega.

[96] Gray K.I., Adeniyi K.O., Zolotovskaya S., Adegoke O. (2025). Rapid and sensitive detection of recombinant BK polyomavirus VP1 protein using a molecularly imprinted impedimetric sensor based on poly(o-phenylenediamine)-ZnTeSe@CoCu core/shell quantum dots modified screen-printed gold electrode. Mater. Sci. Semicon. Proc..

[97] Choi D.Y., Yang J.C., Hong S.W., Park J. (2022). Molecularly imprinted polymer-based electrochemical impedimetric sensors on screen-printed carbon electrodes for the detection of trace cytokine IL-1β. Biosens. Bioelectron..

[98] Sanati A., Siavash Moakhar R., Hosseini I.I., Raeissi K., Karimzadeh F., Jalali M., Kharaziha M., Sheibani S., Shariati L., Presley J.F. (2021). Gold nano/micro-islands overcome the molecularly imprinted polymer limitations to achieve ultrasensitive protein detection. ACS Sens..

[99] Cao S., Xiao G., Liao W., Zhao B., Xie S., Liu Z., Chen W., Yue Z. (2025). Dual-functional monomer-based molecularly imprinted light-addressable photoelectrochemical platform for multichannel detection of molecules with various sizes. ACS Sens..

[100] Ayman Saleh M., Khorrami Jahromi A., Shieh H., Siavash Moakhar R., del Real Mata C., Mahshid S. (2024). A reagentless molecularly imprinted polymer-based electrochemical biosensor for single-step detection of troponin I in biofluids. Analyst.

[101] Adeniyi K.O., Oyinlola K., Achadu O.J., Menard H., Grillo F., Yang Z., Adegoke O. (2024). Molecularly imprinted viral protein integrated Zn–Cu–In–Se–P quantum dots superlattice for quantitative ratiometric electrochemical detection of SARS-CoV-2 spike protein in saliva. ACS Appl. Nano Mater..

[102] Yarman A. (2024). Effect of various carbon electrodes on MIP-based sensing proteins using poly(scopoletin): A case study of ferritin. Biomimetics.

[103] Bosserdt M., Gajovic-Eichelman N., Scheller F.W. (2013). Modulation of direct electron transfer of cytochrome c by use of a molecularly imprinted thin film. Anal. Bioanal. Chem..

[104] Stojanovic Z., Erdőssy J., Keltai K., Scheller F.W., Gyurcsányi R.E. (2017). Electrosynthesized molecularly imprinted polyscopoletin nanofilms for human serum albumin detection. Anal. Chim. Acta.

[105] Moreira B., Calvet S., Marques A., Martins G.V. (2026). Chitosan-based molecularly imprinted polymers for point-of-care C-reactive protein detection. Talanta.

[106] Moreira F.T.C., Sales M.G.F. (2019). Autonomous biosensing device merged with photovoltaic technology for cancer biomarker detection. J. Electroanal. Chem..

[107] Wang Q., Xue R., Guo H., Wei Y., Yang W. (2018). A facile horseradish peroxidase electrochemical biosensor with surface molecular imprinting based on polyaniline nanotubes. J. Electroanal. Chem..

[108] Moreira F.T.C., Sales M.G.F. (2017). Smart naturally plastic antibody based on poly(α-cyclodextrin) polymer for β-amyloid-42 soluble oligomer detection. Sens. Actuators B Chem..

[109] Park R., Jeon S., Lee J.W., Jeong J., Kwon Y.W., Kim S.H., Jang J., Hong S.W. (2023). Mobile point-of-care device using molecularly imprinted polymer-based chemosensors targeting interleukin-1β biomarker. Biosensors.

[110] Lee J.W., Park R., Jeon S., Kim S.H., Han D.-W., Hong S.W. (2025). Rationally designed molecularly imprinted polymer electrochemical biosensor with graphene oxide interface for selective detection of matrix metalloproteinase-8 (MMP-8). Biosensors.

[111] do Prado Ferreira M., Gorla F.A., Segatelli M.G., Ogatta S.F.Y., Figueiredo E.C., Tarley C.R.T. (2025). Development of method for SARS-CoV-2 spike protein determination in saliva samples at fg mL−1 levels using electrochemically controlled preconcentration and molecularly imprinted polypyrrole sensor. Microchem. J..

[112] Ratautaite V., Boguzaite R., Brazys E., Ramanaviciene A., Ciplys E., Juozapaitis M., Slibinskas R., Bechelany M., Ramanavicius A. (2022). Molecularly imprinted polypyrrole based sensor for the detection of SARS-CoV-2 spike glycoprotein. Electrochim. Acta.

[113] Mazouz Z., Mokni M., Fourati N., Zerrouki C., Barbault F., Seydou M., Kalfat R., Yaakoubi N., Omezzine A., Bouslema A. (2020). Computational approach and electrochemical measurements for protein detection with MIP-based sensor. Biosens. Bioelectron..

[114] Zidarič T., Majer D., Maver T., Finšgar M., Maver U. (2023). The development of an electropolymerized, molecularly imprinted polymer (MIP) sensor for insulin determination using single-drop analysis. Analyst.

[115] Dhinesh Kumar M., Karthikeyan M., Sharma N., Raju V., Vatsalarani J., Kalivendi S.V., Karunakaran C. (2022). Molecular imprinting synthetic receptor based sensor for determination of Parkinson’s disease biomarker DJ-1. Microchem. J..

[116] Yazdani Z., Yadegari H., Heli H. (2019). A molecularly imprinted electrochemical nanobiosensor for prostate specific antigen determination. Anal. Biochem..

[117] Costa R., Costa J., Moreira P., Brandão A.T.S.C., Mafra I., Silva A.F., Pereira C.M. (2022). Molecularly imprinted polymer as a synthetic antibody for the biorecognition of hazelnut Cor a 14-allergen. Anal. Chim. Acta.

[118] Liustrovaite V., Ratautaite V., Ramanaviciene A., Ramanavicius A. (2025). Detection of the SARS-CoV-2 nucleoprotein by electrochemical biosensor based on molecularly imprinted polypyrrole formed on self-assembled monolayer. Biosens. Bioelectron..

[119] Ma Y., Liu C., Zeng Q., Wang L.S. (2019). An impedance molecularly imprinted sensor for the detection of bovine serum albumin (BSA) using the dynamic electrochemical impedance spectroscopy. Electroanalysis.

[120] Pakapongpan S., Poo-arporn Y., Ninket S., Poo-arporn R.P. (2024). A disposable electrochemical sensor for amyloid-β42 protein based on molecular imprinted polymers with nitrogen doped carbon dots-graphene nanohybrid. Microchem. J..

[121] Carvalho M., Gomes R.M., Moreira Rocha S., Barroca-Ferreira J., Maia C.J., Guillade L., Correa-Duarte M.A., Passarinha L.A., Moreira F.T.C. (2023). Development of a novel electrochemical biosensor based on plastic antibodies for detection of STEAP1 biomarker in cancer. Bioelectrochemistry.

[122] Liang A., Lv T., Pan B., Zhu Z., Haotian R., Xie Y., Sun L., Zhang J., Luo A. (2024). Dynamic simulation and experimental studies of molecularly imprinted label-free sensor for determination of milk quality marker. Food Chem..

[123] Drobysh M., Ratautaite V., Brazys E., Ramanaviciene A., Ramanavicius A. (2024). Molecularly imprinted composite-based biosensor for the determination of SARS-CoV-2 nucleocapsid protein. Biosens. Bioelectron..

[124] Özcan N., Medetalibeyoglu H., Akyıldırım O., Atar N., Yola M.L. (2020). Electrochemical detection of amyloid-β protein by delaminated titanium carbide MXene/multi-walled carbon nanotubes composite with molecularly imprinted polymer. Mater. Today Chem..

[125] Wang Z., Li F., Xia J., Xia L., Zhang F., Bi S., Shi G., Xia Y., Liu J., Li Y. (2014). An ionic liquid-modified graphene based molecular imprinting electrochemical sensor for sensitive detection of bovine hemoglobin. Biosens. Bioelectron..

[126] Chen H.-J., Zhang Z.-H., Luo L.-J., Yao S.-Z. (2012). Surface-imprinted chitosan-coated magnetic nanoparticles modified multi-walled carbon nanotubes biosensor for detection of bovine serum albumin. Sens. Actuators B Chem..

[127] Dabrowski M., Lach P., Cieplak M., Kutner W. (2018). Nanostructured molecularly imprinted polymers for protein chemosensing. Biosens. Bioelectron..

[128] Lahcen A.A., Rauf S., Aljedaibi A., de Oliveira Filho J.I., Beduk T., Mani V., Alshareef H.N., Salama K.N. (2021). Laser-scribed graphene sensor based on gold nanostructures and molecularly imprinted polymers: Application for Her-2 cancer biomarker detection. Sens. Actuators B Chem..

[129] Li Y., Jiang C. (2018). Trypsin electrochemical sensing using two-dimensional molecularly imprinted polymers on 96-well microplates. Biosens. Bioelectron..

[130] Moreira F.T.C., Sharma S., Dutra R.A.F., Noronha J.P.C., Cass A.E.G., Sales M.G.F. (2014). Protein-responsive polymers for point-of-care detection of cardiac biomarker. Sens. Actuators B Chem..

[131] Pacheco J.G., Silva M.S.V., Freitas M., Nouws H.P.A., Delerue-Matos C. (2018). Molecularly imprinted electrochemical sensor for the point-of-care detection of a breast cancer biomarker (CA 15-3). Sens. Actuators B Chem..

[132] Ferreira N.S., Moreira A.P.T., de Sá M.H.M., Sales M.G.F. (2017). New electrochemically-derived plastic antibody on a simple conductive paper support for protein detection: Application to BSA. Sens. Actuators B Chem..

[133] Nadim A.H., Abd El-Aal M.A., Al-Ghobashy M.A., El-Saharty Y.S. (2021). Facile imprinted polymer for label-free highly selective potentiometric sensing of proteins: Case of recombinant human erythropoietin. Anal. Bioanal. Chem..

[134] Abbasy L., Mohammadzadeh A., Hasanzadeh M., Razmi N. (2020). Development of a reliable bioanalytical method based on prostate specific antigen trapping on the cavity of molecular imprinted polymer towards sensing of PSA using binding affinity of PSA-MIP receptor: A novel biosensor. J. Pharm. Biomed. Anal..

[135] Moreira F.T.C., Dutra R.A.F., Noronha J.P.C., Cunha A.L., Sales M.G.F. (2011). Artificial antibodies for troponin T by its imprinting on the surface of multiwalled carbon nanotubes: Its use as sensory surfaces. Biosens. Bioelectron..

[136] Rebelo T.S.C.R., Santos C., Costa-Rodrigues J., Fernandes M.H., Noronha J.P., Sales M.G.F. (2014). Novel prostate specific antigen plastic antibody designed with charged binding sites for an improved protein binding and its application in a biosensor of potentiometric transduction. Electrochim. Acta.

[137] Kalecki J., Iskierko Z., Cieplak M., Sharma P.S. (2020). Oriented immobilization of protein templates: A new trend in surface imprinting. ACS Sens..

[138] Johari-Ahar M., Karami P., Ghanei M., Afkhami A., Bagheri H. (2018). Development of a molecularly imprinted polymer tailored on disposable screen-printed electrodes for dual detection of EGFR and VEGF using nano-liposomal amplification strategy. Biosens. Bioelectron..

[139] Karami P., Bagheri H., Johari-Ahar M., Khoshsafar H., Arduini F., Afkhami A. (2019). Dual-modality impedimetric immunosensor for early detection of prostate-specific antigen and myoglobin markers based on antibody-molecularly imprinted polymer. Talanta.

[140] Tlili A., Attia G., Khaoulani S., Zerrouki C., Yaakoubi N., Othmane A., Fourati N. (2025). Rethinking the use of redox probes for the detection of electroactive proteins with electrochemical sensors modified with molecularly imprinted polymers. Biosens. Bioelectron..

[141] Tlili A., Attia G., Khaoulani S., Mazouz Z., Zerrouki C., Yaakoubi N., Othmane A., Fourati N. (2021). Contribution to the understanding of the interaction between a polydopamine molecular imprint and a protein model: Ionic strength and pH effect investigation. Sensors.

[142] Tlili A., Ayed D., Attia G., Fourati N., Zerrouki C., Othmane A. (2023). Comparative study of two surface techniques of proteins imprinting in a polydopamine matrix. Application to immunoglobulin detection. Talanta.

[143] Oliveira D., Romaguera Barcelay Y., Moreira F.T.C. (2024). An electrochemically synthesized molecularly imprinted polymer for highly selective detection of breast cancer biomarker CA 15-3: A promising point-of-care biosensor. RSC Adv..

[144] Cieplak M., Szwabinska K., Sosnowska M., Chandra B.K.C., Borowicz P., Noworyta K., D’Souza F., Kutner W. (2015). Selective electrochemical sensing of human serum albumin by semi-covalent molecular imprinting. Biosens. Bioelectron..

[145] Ting W.-T., Wang M.-J., Howlader M.M.R. (2024). Interleukin-6 electrochemical sensor using poly(o-phenylenediamine)-based molecularly imprinted polymer. Sens. Actuators B Chem..

[146] Ting W.-T., Ali M.Y., Mitea V., Wang M.-J., Howlader M.M.R. (2024). Polyaniline-based bovine serum albumin imprinted electrochemical sensor for ultra-trace-level detection in clinical and food safety applications. Int. J. Biol. Macromol..

[147] Liang A., Tang S., Liu M., Yi Y., Xie B., Hou H., Luo A. (2022). A molecularly imprinted electrochemical sensor with tunable electrosynthesized Cu-MOFs modification for ultrasensitive detection of human IgG. Bioelectrochemistry.

[148] Dąbrowski M., Zimińska A., Kalecki J., Cieplak M., Lisowski W., Maksym R., Shao S., D’Souza F., Kuhn A., Sharma P.S. (2019). Facile fabrication of surface-imprinted macroporous films for chemosensing of human chorionic gonadotropin hormone. ACS Appl. Mater. Interfaces.

[149] Di Giulio T., Mazzotta E., Malitesta C. (2020). Molecularly imprinted polyscopoletin for the electrochemical detection of the chronic disease marker lysozyme. Biosensors.

[150] Luo A., Cai Y., Liu M., Tang S., Zhu Z., Haotian R., Xie B., Yi Y., Hao Z., Liang A. (2022). Novel Co MOF with ionic liquid comprised portable molecularly imprinted polymer-based electrochemical sensor for the point-of-care detection of a breast cancer biomarker. J. Electrochem. Soc..

[151] Zhang Z., Chen S., Ren J., Han F., Yu X., Tang F., Xue F., Chen W., Yang J., Jiang Y. (2020). Facile construction of a molecularly imprinted polymer–based electrochemical sensor for the detection of milk amyloid A. Microchim. Acta.

[152] Li L., Yang L., Xing Z., Lu X., Kan X. (2013). Surface molecularly imprinted polymers-based electrochemical sensor for bovine hemoglobin recognition. Analyst.

[153] Ayankojo A.G., Boroznjak R., Reut J., Tuvikene J., Timmusk T., Syritski V. (2023). Electrochemical sensor based on molecularly imprinted polymer for rapid quantitative detection of brain-derived neurotrophic factor. Sens. Actuators B Chem..

[154] Ayankojo A.G., Boroznjak R., Reut J., Öpik A., Syritski V. (2022). Molecularly imprinted polymer based electrochemical sensor for quantitative detection of SARS-CoV-2 spike protein. Sens. Actuators B Chem..

[155] Antipchik M., Reut J., Ayankojo A.G., Öpik A., Syritski V. (2022). MIP-based electrochemical sensor for direct detection of hepatitis C virus via E2 envelope protein. Talanta.

[156] Kidakova A., Boroznjak R., Reut J., Öpik A., Saarma M., Syritski V. (2020). Molecularly imprinted polymer-based SAW sensor for label-free detection of cerebral dopamine neurotrophic factor protein. Sens. Actuators B Chem..

[157] Yin Z.-Z., Liu Z., Zhou M., Yang X., Zheng G., Zhang H., Kong Y. (2023). A surface molecularly imprinted electrochemical biosensor for the detection of SARS-CoV-2 spike protein by using Cu_7_S_4_-Au as built-in probe. Bioelectrochemistry.

[158] Chai R., Wang Y., Kan X. (2021). Sensitive and selective detection of glycoprotein based on dual-signal and dual-recognition electrochemical sensing platform. Food Chem..

[159] Fu H., Bai Z., Li P., Feng X., Hu X., Song X., Chen L. (2023). Molecular imprinted electrochemical sensor for ovalbumin detection based on boronate affinity and signal amplification approach. Food Chem..

[160] Huang J., Wu Y., Cong J., Luo J., Liu X. (2018). Selective and sensitive glycoprotein detection via a biomimetic electrochemical sensor based on surface molecular imprinting and boronate-modified reduced graphene oxide. Sens. Actuators B Chem..

[161] Yang X., Liu Z., Kong Y., Yin Z.-Z., Zheng G., Zhang H. (2024). An anti-fouling surface molecularly imprinted ratiometric electrochemical biosensor for SARS-CoV-2 spike protein. Microchem. J..

[162] Chen F., Lv C., Xing Y., Luo L., Wang J., Cheng Y., Xie X. (2023). Electrospinning carbon fibers based molecularly imprinted polymer self-supporting electrochemical sensor for sensitive detection of glycoprotein. Sens. Actuators B Chem..

[163] Liu Z., Yin Z.-Z., Cai W., Wu D., Li J., Kong Y. (2022). A surface protein−imprinted biosensor based on boronate affinity for the detection of anti−human immunoglobulin G. Microchim. Acta.

[164] Kong X., Zhang Y., Liu Y., Lyu J., Yin Z.-Z. (2025). An electrochemical microsensor for osteopontin based on a molecularly imprinted layer and a built-in probe-functionalized acupuncture needle. Anal. Methods.

[165] You M., Yang S., Tang W., Zhang F., He P.-G. (2017). Ultrasensitive electrochemical detection of glycoprotein based on boronate affinity sandwich assay and signal amplification with functionalized SiO_2_@Au nanocomposites. ACS Appl. Mater. Interfaces.

[166] Li Y., Hong M., Miaomiao, Bin Q., Lin Z., Cai Z., Chen G. (2013). Novel composites of multifunctional Fe_3_O_4_@Au nanofibers for highly efficient glycoprotein imprinting. J. Mater. Chem. B.

[167] Sun X., Jian Y., Wang H., Ge S., Yan M., Yu J. (2019). Ultrasensitive microfluidic paper-based electrochemical biosensor based on molecularly imprinted film and boronate affinity sandwich assay for glycoprotein detection. ACS Appl. Mater. Interfaces.

[168] Yang J.C., Lim S.J., Cho C.H., Hazarika D., Park J.P., Park J. (2023). Determination of tumor necrosis factor-α in serum using extended-gate field-effect transistor-based chemosensors with molecularly imprinted polymer-coated gold dendrites. Sens. Actuators B Chem..

[169] Ferreira B., Correa-Duarte M., Marques A., Moreira F., Martins G. (2024). Bioinspired host-tailored polymers based on molecular imprinting for cytokine assessment. Microchem. J..

[170] Li W., Zhang Q., Wang Y., Ma Y., Guo Z., Liu Z. (2019). Controllably prepared aptamer–molecularly imprinted polymer hybrid for high-specificity and high-affinity recognition of target proteins. Anal. Chem..

[171] Jolly P., Tamboli V., Harniman R.L., Estrela P., Allender C.J., Bowen J.L. (2016). Aptamer–MIP hybrid receptor for highly sensitive electrochemical detection of prostate specific antigen. Biosens. Bioelectron..

[172] Khumsap T., Corpuz A., Nguyen L.T. (2021). Epitope-imprinted polymers: Applications in protein recognition and separation. RSC Adv..

[173] Singhal A., Singh A., Shrivastava A., Khan R. (2023). Epitope imprinted polymeric materials: Application in electrochemical detection of disease biomarkers. J. Mater. Chem. B.

[174] Dechtrirat D., Jetzschmann K.J., Stöcklein W.F.M., Scheller F.W., Gajovic-Eichelmann N. (2012). Protein rebinding to a surface-confined imprint. Adv. Funct. Mater..

[175] Zhang X., Caserta G., Yarman A., Supala E., Waffo A., Wollenberger U., Gyurcsányi R., Zebger I., Scheller F. (2021). “Out of pocket” protein binding—A dilemma of epitope imprinted polymers revealed for human hemoglobin. Chemosensors.

[176] Tchinda R., Tutsch A., Schmid B., Süssmuth R.D., Altintas Z. (2019). Recognition of protein biomarkers using epitope-mediated molecularly imprinted films: Histidine or cysteine modified epitopes?. Biosens. Bioelectron..

[177] Li M.-X., Wang X.-H., Zhang L.-M., Wei X.-P. (2017). A high sensitive epitope imprinted electrochemical sensor for bovine serum albumin based on enzyme amplifying. Anal. Biochem..

[178] Yarman A., Waffo A.F.T., Katz S., Bernitzky C., Kovács N., Borrero P., Frielingsdorf S., Supala E., Dragelj J., Kurbanoglu S. (2024). A strep-tag imprinted polymer platform for heterogenous bio(electro)catalysis. Angew. Chem. Int. Ed..

[179] Shao H., Liu Z. (2024). Epitope imprinted electrochemical sensor for highly sensitive detection of alpha-fetoprotein. Electrochim. Acta.

[180] Wang X., Deng H., Wang C., Wei Q., Wang Y., Xiong X., Li C., Li W. (2020). A pro-gastrin-releasing peptide imprinted photoelectrochemical sensor based on the in situ growth of gold nanoparticles on a MoS_2_ nanosheet surface. Analyst.

[181] Drzazgowska J., Schmid B., Süssmuth R.D., Altintas Z. (2020). Self-assembled monolayer epitope bridges for molecular imprinting and cancer biomarker sensing. Anal. Chem..

[182] Altintas Z., Takiden A., Utesch T., Mroginski M.A., Schmid B., Scheller F.W., Süssmuth R.D. (2019). Integrated approaches toward high-affinity artificial protein binders obtained via computationally simulated epitopes for protein recognition. Adv. Funct. Mater..

[183] Zhang X., Yarman A., Kovács N., Bognár Z., Gyurcsányi R.E., Bier F.F., Scheller F.W. (2024). Specific features of epitope-MIPs and whole-protein MIPs as illustrated for AFP and RBD of SARS-CoV-2. Microchim. Acta.

[184] Bognár Z., Supala E., Yarman A., Zhang X., Bier F.F., Scheller F.W., Gyurcsányi R.E. (2022). Peptide epitope-imprinted polymer microarrays for selective protein recognition. Application for SARSCoV-2 RBD protein. Chem. Sci..

[185] Zhang X., Waffo A.T., Yarman A., Kovács N., Bognár Z., Wollenberger U., El-Sherbiny I.M., Hassan R.Y.A., Bier F.F., Gyurcsányi R.E. (2022). How an ACE2 mimicking epitope-MIP nanofilm recognizes template-related peptides and the receptor binding domain of SARS-CoV-2. Nanoscale.

[186] Singh M., Srivastava A., Mandal M. (2025). Unravelling the potential of zwitterionic polymers in molecular imprinting. Langmuir.

[187] Zhang X., Du X., Huang X., Lv Z. (2013). Creating protein-imprinted self-assembled monolayers with multiple binding sites and biocompatible imprinted cavities. J. Am. Chem. Soc..

[188] Zhang X., Du X. (2020). Creation of glycoprotein imprinted self-assembled monolayers with dynamic boronate recognition sites and imprinted cavities for selective glycoprotein recognition. Soft Matter.

